# Constructing germline research cohorts from the discarded reads of clinical tumor sequences

**DOI:** 10.1186/s13073-021-00999-4

**Published:** 2021-11-08

**Authors:** Alexander Gusev, Stefan Groha, Kodi Taraszka, Yevgeniy R. Semenov, Noah Zaitlen

**Affiliations:** 1 Division of Population Sciences, Dana-Farber Cancer Institute and Harvard Medical School, Boston, MA USA; 2 Division of Genetics, Brigham and Women’s Hospital and Harvard Medical School, Boston, MA USA; 3 The Broad Institute of MIT & Harvard, Cambridge, MA USA; 4 Departments of Neurology and Computational Medicine, UCLA, Los Angeles, CA USA; 5 Department of Dermatology, Massachusetts General Hospital, Boston, MA USA

## Abstract

**Background:**

Hundreds of thousands of cancer patients have had targeted (panel) tumor sequencing to identify clinically meaningful mutations. In addition to improving patient outcomes, this activity has led to significant discoveries in basic and translational domains. However, the targeted nature of clinical tumor sequencing has a limited scope, especially for germline genetics. In this work, we assess the utility of discarded, off-target reads from tumor-only panel sequencing for the recovery of genome-wide germline genotypes through imputation.

**Methods:**

We developed a framework for inference of germline variants from tumor panel sequencing, including imputation, quality control, inference of genetic ancestry, germline polygenic risk scores, and HLA alleles. We benchmarked our framework on 833 individuals with tumor sequencing and matched germline SNP array data. We then applied our approach to a prospectively collected panel sequencing cohort of 25,889 tumors.

**Results:**

We demonstrate high to moderate accuracy of each inferred feature relative to direct germline SNP array genotyping: individual common variants were imputed with a mean accuracy (correlation) of 0.86, genetic ancestry was inferred with a correlation of > 0.98, polygenic risk scores were inferred with a correlation of > 0.90, and individual HLA alleles were inferred with a correlation of > 0.80. We demonstrate a minimal influence on the accuracy of somatic copy number alterations and other tumor features. We showcase the feasibility and utility of our framework by analyzing 25,889 tumors and identifying the relationships between genetic ancestry, polygenic risk, and tumor characteristics that could not be studied with conventional on-target tumor data.

**Conclusions:**

We conclude that targeted tumor sequencing can be leveraged to build rich germline research cohorts from existing data and make our analysis pipeline publicly available to facilitate this effort.

**Supplementary Information:**

The online version contains supplementary material available at 10.1186/s13073-021-00999-4.

## Background

Large-scale tumor sequencing is ubiquitous in the clinical setting, and hundreds of thousands of cancer patients have had tumors sequenced on targeted panels in order to identify clinically actionable mutations [1–5]. Data from these cohorts provide an unprecedented opportunity for basic research and translational discovery, improving our understanding of cancer biology and supporting clinical decision-making. Recent FDA approval of such technologies across many cancer types will likely lead to even broader adoption in the coming years [6].

Studies of somatic alterations from targeted tumor sequencing have characterized drivers of tumor evolution that can often influence treatment response and outcomes [2, 7–10]. However, the majority of clinical sequencing platforms do not collect germline data and focus only on exons within a small number of known cancer genes [1, 2], thereby excluding potentially meaningful germline variation and the 98% of the genome that is non-coding. For example, the American Association for Cancer Research Project GENIE (Genomics Evidence Neoplasia Information Exchange) has aggregated sequence data on > 100,000 tumors from 19 international cancer centers with the aim of incorporating clinical outcomes [11]. However, 17/19 participating institutions use tumor-only sequencing with no matched normal specimen, and the average sequencing panel in the study covers fewer than 250 genes. Commercial genomic platforms have similarly sequenced hundreds of thousands of patients on targeted, tumor-only panels of limited scope [12]. In contrast to targeted sequencing, tumor/normal matched whole-genome sequencing (WGS) provides a comprehensive cancer genomics assay [13–16], but sequencing costs have greatly limited the sample size and power of these studies. The ability to leverage genome-wide germline information from the wealth of the existing and ongoing targeted tumor data would broaden the scope of feasible research and offer opportunities to incorporate germline genetics into existing clinical workflows.

Recent work has shown that low-coverage sequencing can be used to accurately impute common germline polymorphisms by leveraging linkage disequilibrium (LD) information within the low-coverage data [17–23]. However, such approaches have largely been applied to sequencing of blood/normal tissue and have not been benchmarked for tumor data, nor for data with the severe coverage variation of targeted panels, which can often yield > 100× on-target and < 0.1× off-target coverage. Here, we demonstrate that similar techniques can be used to infer common germline variation from targeted sequencing of tumors. We first use extensive benchmarking with real tumor/germline data to show that off-target tumor sequencing can be used to accurately estimate common germline genotypes. We then aggregate these genotypes to infer genetic ancestry, polygenic risk scores (PRS), and HLA alleles and demonstrate high to moderate imputation accuracy of each. Finally, we showcase the research utility of this data by identifying associations with germline risk and genetic ancestry in a “real world” cohort of > 25,000 tumors.

## Methods

### Overview of data

We collected 25,889 tumors spanning > 20 cancer types as part of the Dana-Farber PROFILE cohort, which were prospectively sequenced on the OncoPanel platform as part of routine cancer care (Additional file 1: Fig. S1). A benchmarking study of somatic variation using 3700 cases from this cohort was previously carried out [24] though no germline analyses have been published prior to the current work. The OncoPanel platform targeted the exons of 275–447 cancer genes on one of three panel versions, as well as a fourth panel “subversion” (referred to as panel “3.1”) that modified the sequencing chemistry but not the targeted exons [1, 24]. Genomewide, the mean sequencing coverage was 0.036× (ranging from 0.022 to 0.043× across the panel versions), compared to a mean on-target coverage of 152× (Additional file 1: Fig. S2). A subset of 833 individuals had DNA available from the whole blood and was genotyped on the Illumina Multi-Ethnic Genotyping Array (MEGA) and used for benchmarking. Written informed consent was obtained from participants prior to inclusion in this study.

### Patient consent, accrual, and tumor sequencing

PROFILE samples were selected and sequenced from patients who were consented under institutional review board (IRB)-approved protocol 11-104 and 17-000 from the Dana-Farber/Partners Cancer Care Office for the Protection of Research Subjects. Written informed consent was obtained from participants prior to inclusion in this study. Secondary analyses of previously collected data were performed with approval from the Dana-Farber IRB: DFCI IRB protocol 19-033 and 19-025; waiver of Health Insurance Portability and Accountability Act (HIPAA) authorization approved for both protocols.

Patients were recruited based on available material and consent and were not otherwise ascertained for age, sex, stage, or tumor site. Eighty-nine percent of samples were formalin-fixed paraffin-embedded (FFPE), which is the standard clinical workflow (both in our cohort and across many institutions [2, 11]), with the remainder being an incidental combination of samples from blood, fresh frozen tissue, or bone marrow. Quantification of *somatic* calling from FFPE versus non-FFPE has been carried out previously [1]. Each sample was sequenced on one of three panel versions targeting the exons of 275, 300, and 447 genes, respectively [1, 24]. Sequencing was performed using an Illumina HiSeq 2500 with 2 × 100 paired-end reads. Samples met a minimum of 30× coverage for 80% of targets for analysis. “On-target” coverage was defined by counting reads overlapping all 1000 Genomes polymorphisms in the targeted regions with > 50× reads; “off-target” coverage was defined using reads overlapping all 1000 Genomes polymorphisms in the rest of the genome.

### Germline genotyping

A subset of 833 patients with tumor sequencing was also germline genotyped as part of the Mass General Brigham Biobank. DNA samples were processed from the whole blood and genotyped on either the Illumina Multi-Ethnic Genotyping Array (MEGA), the Expanded Multi-Ethnic Genotyping Array (MEGA Ex) array, or the Multi-Ethnic Global (MEG) BeadChip [25]. All germline samples were imputed to the Haplotype Reference Consortium (HRC) reference panel [26] and then restricted to ~ 1.1 million HapMap3 variants that typically exhibit high imputation accuracy across genotyping platforms and uniformly tag common SNP variation [27]. Small indels were not available in the HRC reference panel due to sequencing ambiguity, and we additionally imputed small indels into the germline genotyped data using the 1000 Genomes Phase 3 reference panel [28] and restricted to high-quality indels with INFO score (imputation confidence score) > 0.9.

### Germline imputation from tumors

We assessed three imputation algorithms intended for low-coverage data: STITCH v1.5.3 [21], GLIMPSE v1.0.0 [29], and QUILT v0.1.9 [30]. For all analyses, OncoPanel data was aligned to hg19 using bwa and processed with the GATK IndelRealigner. The 1000 Genomes Phase 3 release was used as a haplotype reference, targeting variants with > 1% frequency in the European population. Tumor imputation was performed using the 1000 Genomes reference (rather than the HRC reference) because the HRC panel is not publicly available and the HRC imputation server does not support raw sequencing data. We thus sought to use the best reference panels that were accessible for the two data types. We note that HRC largely improves imputation accuracy for low-frequency variants [26], which were not the target of our analysis.

Imputation with STITCH was carried out on all samples using aligned reads in 5-MB batches (see the “Availability of data and materials” section for the detailed parameters and code). The potential influence of target cohort size was evaluated by randomly downsampling to a lower number of sequenced tumors. Imputation with QUILT was carried out using the same input and batching procedure, with default parameters. Imputation with GLIMPSE was carried out on all samples with default parameters as recommended in the documentation: calling genotype likelihoods from each raw BAM file, splitting the genome into chunks, performing imputation and phasing, and ligating the chunks. An alternative, reference-only version of GLIMPSE was kindly provided to us by the authors but could not be compiled in our computing environment. Lastly, we considered two other imputation approaches: GeneImp [31] and BEAGLE [32], but found that their computational requirements were infeasible for sample sizes in the thousands. Identical reference panel data was used for all methods except small indels, structural variants, and multi-allelic polymorphisms were excluded from the STITCH and GLIMPSE analysis (which only allows biallelic single nucleotides). After imputation, variants were considered “filtered” if they had a minor allele frequency > 1% and an INFO score (imputation confidence score) > 0.4 (similar to parameters used previously [20]). Additional filtering thresholds were investigated in Additional file 1: Fig. S3.

### Quantifying imputation accuracy

All analyses imputed the allele “dosage” for each individual and site, defined as the expected number of non-reference alleles carried by the individual, and accuracy was estimated using two metrics: Pearson correlation and allelic error. First, Pearson correlation was computed for each imputed polymorphism across all individuals between the tumor imputed and germline variant dosages. Pearson correlation (or squared correlation) has been commonly used to evaluate variant imputation in prior work [26, 29, 30] and can be interpreted as the effective reduction in sample size for an association statistic (i.e., a variant with an imputation correlation of 0.85 is expected to have the statistical power of a directly genotyped study with 0.85^2^ = 0.72 times the size) [33]. We confirmed, by random downsampling, that the mean Pearson correlation was not biased by the number of variants included and that the mean Pearson correlation was 0 (as expected) when applied to random samples (Additional file 1: Fig. S4). Second, allelic error was computed as the difference between the imputed and the genotyped allele (relative to the human reference allele) and summarized as either mean allelic error (to quantify any reference-specific bias) or average absolute error.

### Quantifying somatic copy number alterations

Local copy number was called by the default analysis pipeline used for clinical reporting to patients and physicians. The RobustCNV (v2.0.1) algorithm was applied to individual tumors along with a panel of normals to identify copy number segments based on coverage [34]. We then identified the 5% of individual-segment pairs with the highest and lowest estimated segment mean in the population and computed imputation accuracy within these individual-segment pairs or for all other (“neutral”) regions.

### Quantifying somatic copy-neutral loss of heterozygosity (CN-LOH)

In addition to somatic copy number alterations that are observable through decreased/increased coverage, we sought to identify regions of somatic CN-LOH. Robust detection of CN-LOH from tumor-only panel sequencing remains a challenge and CN-LOH is not called by our in-house somatic pipeline. Rather than attempt to unambiguously call all CN-LOH regions in all samples, we instead focused on the most extreme/likely CN-LOH regions (akin to our previous analyses of the 5% deepest amplifications/deletions). We reasoned that CN-LOH would be detectable at germline heterozygous variants with high coverage but extreme allelic imbalance in the tumor, indicative of loss/gain of a single allele [35]. For each sample, we identified high-coverage, on-target variants in the tumor that overlapped germline heterozygous variants in the corresponding SNP array, to be used as indicators of somatic allelic imbalance. For each such heterozygote, we quantified the number of somatic reads mapping to the two alleles in the tumor, restricted to high-coverage variants with > 50 reads and > 5 reads for each allele (to avoid false positives from homozygous sites that were genotyped as heterozygous in error), and quantified the distribution of allelic fractions. We then identified the top 5% of sites with the most substantial allelic imbalance deviation from 50:50 (corresponding to an allele fraction of either 0.0–0.23 or 0.77–1.0) as putative CN-LOH (Additional file 1: Fig. S5a). These sites had slightly lower mean coverage relative to other sites, consistent with our goal of copy-neutrality (Additional file 1: Fig. S5b). Finally, we expanded each site by 100 kb to define CN-LOH “regions.”

### Quantifying somatic SNVs in whole-genome data

We used data from 25 cancers from the PCAWG consortium [16] to quantify the expected number of somatic single nucleotide variants (SNVs) overlapping a population SNP in the reference panel. Release v28 somatic SNV positions were downloaded from the PCAWG/ICGC data portal for each cancer type. The somatic SNVs in each tumor were then overlapped with the 7,568,773 common SNPs that were targeted for germline imputation and quantified.

### Germline HLA allele inference

HLA alleles were inferred from the imputed germline genotypes using the SNP2HLA pipeline [36] and the NIDDK HLA reference panel with default parameters [37]. The NIDDK reference panel contained 5225 samples with serotyped HLA alleles spanning HLA A, B, C, DPA1, DPB1, DQA1, DQB1, and DRB1. Restricting to alleles with at least 1% carriers, the panel contained 83 2-digit alleles and 112 4-digit alleles. We did not find that additional SNP exclusion or quality control prior to HLA inference produced any measurable improvement in accuracy and thus used all imputed variants retained after the first post-imputation filtering step. After HLA imputation, we defined a set of “filtered” HLA variants with an INFO score (imputation confidence score) > 0.4 and at least one call with probability > 0.5.

HLA imputation yielded an estimate of carrier and non-carrier status for each allele, which does not map directly to homozygosity due to the presence of multiple alleles per locus. HLA homozygosity (*h*) was computed for each individual and locus (A, B, C, and D*) as follows:

$$ {h}_A={\sum}_{\mathrm{alleles}\ \mathrm{a}\ \mathrm{in}\ \mathrm{locus}\ A}{p}_a{\prod}_{\mathrm{alleles}\ \mathrm{b}\ne \mathrm{a}\ \mathrm{in}\ \mathrm{locus}\ A}{q}_b $$

where *p*_*a*_ is the probability of being a homozygous carrier for allele *a*, and *q*_*b*_ is the probability of being a non-carrier of allele *b*. The probability of being homozygous for at least one locus was then computed across loci as follows:

$$ {h}_{1+}=1-{\prod}_{\mathrm{loci}\ A}\left(1-{h}_A\right). $$

with separate computations of *h*_1+_ for the MHC class I and class II alleles.

### Polygenic risk score inference

Publicly available GWAS data from studies of cancer and related traits was used to compute polygenic risk scores (PRS). Studies used included breast cancer [38], glioma [39], non-small cell lung cancer [40], ovarian cancer [41], prostate cancer [42], and melanoma [43] from case-control GWAS data, as well as smoking, skin pigmentation, and tanning ability from the UK Biobank cohort analysis [44]. GWAS SNPs were restricted to HapMap3 SNPs, which are typically well imputed and thoroughly capture common SNP variation.

For evaluating tumor versus germline PRS inference accuracy, all available SNPs were included and no additional LD pruning/clumping or *p*-value thresholding was applied, to minimize any parameter tuning. For each trait and individual, a PRS was constructed as the sum of allele dosages weighted by the GWAS association statistic using either (a) off-target imputed SNPs or (b) genotype array SNPs as the gold standard. Accuracy was quantified using the slope and correlation from a linear regression of the off-target score on the gold-standard score.

For evaluating the association with the target cancer type, LD pruning was applied to genome-wide significant (*p* < 5 × 10^−8^) HapMap3 SNPs from each GWAS study, and the PRS was computed as the weighted sum described above. Association was estimated in a logistic regression with the quantitative score as the independent variable and the dependent variable defined as having a tumor from the target cancer versus any other cancer, with sex, age, and panel version included as a covariate. For computing odds ratios (ORs) for individuals with high PRS, the quantitative score was replaced with an indicator for the top decile of the PRS in the same logistic regression.

### Genetic ancestry inference

Samples were projected into genetic ancestry principal components using the weights previously derived by the SNPWEIGHTS software [45] for the continental populations. Weights were constructed from the 1000 Genomes reference groups with ancestry from Northern/Western Europe (CEU), Western Africa (YRI), and China (CHB+CHD). In our data, each component was projected independently as a linear combination of the weights and individual sample dosages (using the plink2 “--score” command). Components were then linearly recalibrated by fitting to self-reported race as an outcome (note this linear recalibration is for interpretation purposes only and does not influence the significance of any downstream associations). To estimate ancestry fractions, we uniformly rescaled the African and Asian components to be between 0 and 1 and additionally uniformly scaled the ancestry of each individual to be between 0 and 1.

### Analysis of *EGFR* mutation carriers in non-small cell lung cancer (NSCLC)

We restricted to 2900 NSCLC samples from the full cohort and quantified carrier status for somatic SNVs in the *EGFR* gene. All samples targeted *EGFR* for sequencing. Somatic variants were called using the default analysis pipeline used for clinical reporting: the MuTect algorithm [46] with a panel of normals followed by filtering of any common variants in the Gnomad reference panel [47]. Only non-synonymous variants in *EGFR* exons were retained and being a carrier was defined as having > 0 mutations. Carrier status was associated with genetic ancestry using logistic regression with covariates for sex, age, tumor purity, and panel versions.

## Results

### Accurate inference of common germline genotypes from tumor-only sequencing

**Fig. 1:**
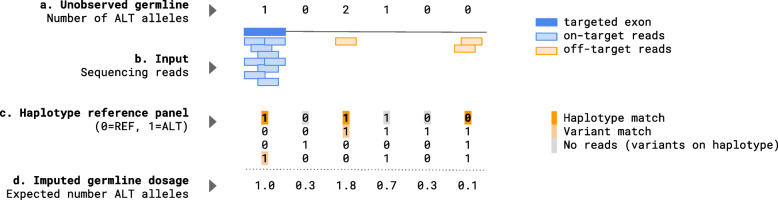
Schematic of germline imputation from low-coverage sequencing. **a** The unobserved germline variants to be imputed, with 0/1/2 reflecting the number of ALT (non-reference) alleles. **b** The sequenced input data with targeted regions (dark blue), on-target reads aligning to these regions (light blue), and off-target reads typically discarded (orange). **c** The haplotype reference panel as a matrix of 0/1 alleles (corresponding to reference or ALT). Alleles that match sequenced reads are shown in light orange, alleles that form a haplotype match shown in dark orange, and alleles that reside along the haplotype but did not carry reads in the target sample shown in gray. **d** The imputed germline variant dosages/probabilities, quantifying the expected number of non-reference alleles. In sum, the matched haplotype is used to refine the dark orange alleles and impute the light gray alleles in the target individual

Common germline genotypes were imputed directly from off-target tumor sequencing reads using the 1000 Genomes reference panel and evaluated against the gold standard germline SNP genotyping (Fig. 1, Additional file 1: Fig. S6; see the “Methods” section). We evaluated multiple imputation approaches [29, 30] (see the “Methods” section) and found that the STITCH algorithm, developed for reference-free imputation, yielded the highest overall accuracy (mean Pearson correlation = 0.79 s.e. 0.001) while scaling to tens of thousands of samples (Additional file 1: Fig. S6). All methods exhibited increased imputation accuracy for higher confidence variants (e.g., higher INFO score, which quantifies the confidence of the imputation at each variant) as well as at high coverage sites (Additional file 1: Fig. S7), suggesting that their broad modeling assumptions were met. We additionally benchmarked STITCH across randomly subsampled target individuals, finding that imputation accuracy increased with more target data but achieved diminishing returns at 5000 target individuals (Additional file 1: Fig. S6). We note that none of the evaluated imputation algorithms was intended for tumor panel sequencing, and their performance differences should not be interpreted as an indicator of their performance on more conventional, uniform low-coverage sequencing of normal tissues.

**Fig. 2:**
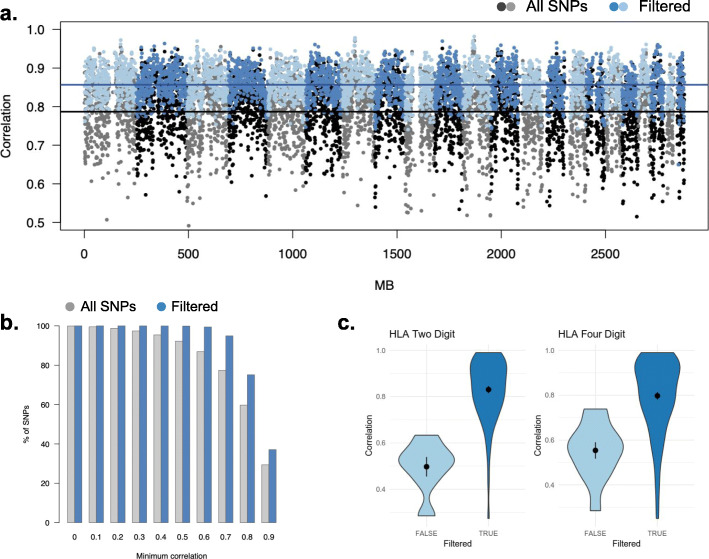
Germline imputation accuracy from tumor sequences. **a** Mean imputation accuracy (Pearson correlation; *y*-axis) across sliding windows of 200 SNPs shown for all variants (gray/black) and filtered variants (INFO > 0.4 and MAF > 1%) (blue/light blue) with alternating shading for chromosomes. **b** Fraction of variants (*y*-axis) as a function of minimum imputation accuracy (Pearson correlation; *x*-axis) for all variants (gray) and filtered variants (blue). **c** Distribution of imputation accuracy (Pearson correlation) for 2-digit and 4-digit HLA alleles that passed (dark blue) or failed (light blue, ~ 10% of variants) INFO-score filtering

Tumor imputed variants from STITCH exhibited a high to moderate correlation with the true germline variant across the entire genome, particularly after basic filtering on imputation confidence (Fig. 2). Mean Pearson correlation was 0.79 (s.d. 0.17) across all 1.1 million SNPs and increased to 0.86 (s.d. 0.087) when restricting to 927,436 “filtered” variants that had an INFO score (imputation confidence) > 0.4 and minor allele frequency > 1% (Fig. 2a). We saw little difference in the mean correlation when restricting to directly genotyped germline SNPs (mean of 0.77 before filtering). A total of 37% of INFO > 0.4 SNPs had a correlation > 0.9 and < 0.5% exhibited a correlation of < 0.6 (compared to 13% of all SNPs exhibiting a correlation of < 0.6) (Fig. 2b; Additional file 1: Fig. S8). INFO score filtering, which did not rely on knowledge of the germline genotypes, thus removed primarily low accuracy SNPs, and we restricted to filtered variants for the remainder of our analyses. Filtering on other parameters did not substantially impact accuracy (Additional file 1: Fig. S3). We note that our analyses excluded small indels, as indel calling from low-coverage sequencing can be unreliable and STITCH does not implement indel imputation. Our attempts to impute indels with other methods or as pseudo-markers did not yield accurate results (Additional file 1: Fig. S9, Additional file 1: Fig. S10), and we believe the problem of high-quality indel imputation (on par with SNPs) remains open.

We investigated the influence of various technical factors on overall correlation and individual-level imputation accuracy (see the “Methods” section). Imputation correlation (post-filtering) was uniform across the genome on all four panel versions (Additional file 1: Fig. S11), ranging from 0.79 to 0.88 with similar trends using individual-level allelic error (Additional file 1: Fig. S12). Surprisingly, imputation error did not track monotonically with the number of genes on each panel, suggesting that coverage and sequencing dynamics play a more important role than the number of targeted exons. At the individual level, imputation error tracked monotonically with coverage across all four panel versions (Additional file 1: Fig. S13). In total, panel version and coverage explained > 80% of the variance in per-individual imputation accuracy (Additional file 1: Fig. S14, Additional file 1: Table S1). Beyond these factors, tumor purity was nominally associated with accuracy (*p* = 1.2 × 10^−3^) but explained negligible variance; neither tumor mutational burden (TMB), primary/metastatic status, nor tissue origin (FFPE versus non-FFPE, see the “Methods” section) were significantly associated with accuracy (Additional file 1: Table S1, Additional file 1: Fig. S15). We sought to stratify tumor purity to evaluate the hypothetical performance of this approach in panel sequencing from normal/non-tumor samples, reasoning that very low purity samples (< 20%) are comparable to normals. However, we observed no significant difference in the accuracy between very low purity samples and very high purity samples (Additional file 1: Fig. S16), consistent with the weak association of continuous purity and accuracy seen above. We thus conclude that technical factors (coverage and sequencing chemistry), rather than tumor-intrinsic confounders, are the primary global drivers of differences in accuracy across individuals.

### Minimal influence of somatic copy number alterations on local imputation accuracy

**Fig. 3:**
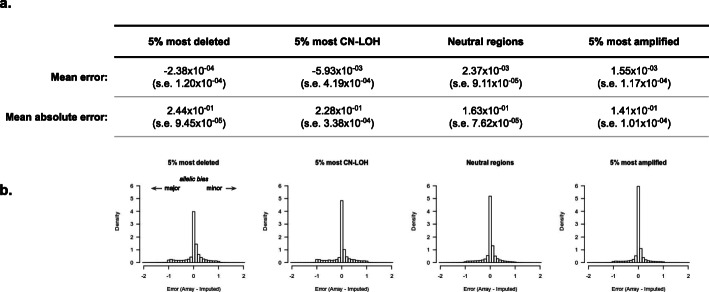
Imputation accuracy for somatically deleted or amplified regions. **a** Table of the mean error and mean absolute error for each region type: 5% most somatically deleted regions; 5% of regions with the greatest copy neutral loss of heterozygosity (CN-LOH), neutral regions (not identified in any other group); and 5% most somatically amplified regions. For each entry, the standard error is reported in parentheses. **b** Histogram of the allelic imputation error between the array ground truth and the imputed dosage; − 1 reflects bias toward the major allele, and + 1 reflects bias toward the minor allele

Tumor genomes often harbor extensive somatic alterations that have the potential to influence local imputation accuracy. We investigated the relationship between somatic copy number alterations (SCNAs) and imputation accuracy by quantifying variant accuracy for the 5% most strongly deleted and the 5% most strongly amplified segments in this cohort (see the “Methods” section). As correlation can be highly uncertain (or incalculable) when computed over a small number of individuals, we focused on the allelic error metrics for this analysis. Allelic error was always computed relative to the major allele to capture any systematic directional biases in the imputation. Surprisingly, for the 5% most amplified regions, absolute error decreased relative to the rest of the genome (more sites imputed with zero error) with no visible artifacts (Fig. 3). This was consistent with amplified regions having higher coverage and thus more reads for the imputation scaffolding, without degrading accuracy. For the most deleted regions, error increased (fewer sites imputed with zero error) and imputed variants exhibited a small but statistically significant bias toward the major allele (Fig. 3, Additional file 1: Fig. S17). This again was consistent with deleted regions exhibiting lower coverage and fewer reads for imputation. In sum, extreme SCNAs had a small influence on imputation error, with deletions leading to lower accuracy and a slight bias toward the major allele.

We additionally investigated imputation accuracy at regions with copy-neutral loss of heterozygosity (CN-LOH) where one haplotype is deleted and the other is amplified, thus producing no loss in coverage. We used somatic allelic imbalance at high coverage heterozygous variants to identify the 5% most imbalanced putative CN-LOH regions (see the “Methods” section; Additional file 1: Fig. S5) and quantified the allelic accuracy within as we had done with deletions and amplifications (Fig. 3, Additional file 1: Fig. S17). Interestingly, the mean imputed allele in CN-LOH regions was slightly but significantly shifted toward the major allele (more strongly than for deletions). On the other hand, the mean absolute imputation error was slightly but significantly higher than average (but less strongly than for deletions). In sum, we found that the most extreme CN-LOH regions operate akin to the most extreme deletions, but with a stronger effect on the mean error and a weaker effect on the absolute error.

Finally, we investigated the potential influence of somatic single nucleotide variants (SNVs) on imputation quality. Out of 38,711 somatic variants with > 1 carrier in our entire tumor cohort, only 30/38,711 (0.08%) were in the imputation reference panel and had a population frequency > 1% such that imputation was attempted. For these 30 variants, the mean imputation INFO score was 0.83 (s.e. 0.02), compared to the mean INFO of 0.67 for an average variant, indicating that they do not suffer from increased imputation uncertainty in the population. We additionally observed no significant differences in mean accuracy between tumors with high/low TMB (Additional file 1: Fig. S15). As SNVs in off-target regions could also, in principle, bias the imputation, we used tumor whole-genome sequencing from the PCAWG consortium [16] to quantify the expected overlap with variants in our reference panel across diverse tumors (see the “Methods” section). We found that an average sample contained 0.16 genome-wide SNVs that overlapped with the reference panel (i.e., much less than a single mutation), with the maximum across cancers being 1.51 SNVs (Additional file 1: Table S2). As variants that are not in the imputation reference panel are effectively ignored for the purpose of imputation, we thus conclude that somatic SNVs are not sufficiently common to influence imputation accuracy even in high TMB individuals.

### Imputation of germline HLA alleles

Genetic variation at the HLA locus has been associated with multiple cancer-related phenotypes [15, 48–50], and we investigated the ability of tumor imputed variants to recover HLA alleles. Importantly, HLA genes were not targeted directly by any of the panels so all inference was based on off-target polymorphisms. A conventional HLA imputation algorithm was used to infer the germline HLA alleles from a reference panel of eight common class I and class II genes [36] using the tumor imputed variants as input (see the “Methods” section). As before, we benchmarked against an independent imputation performed from the germline SNP array data. The mean imputation correlation was 0.80 (two digits) and 0.77 (four digits) across all variants. Filtering based on the INFO score removed ~ 10% of generally low accuracy variants and further increased the imputation correlation to 0.83 (two digits) and 0.80 (four-digit) (Fig. 2c). Lastly, we used the tumor-imputed alleles to estimate whether an individual is homozygous for at least one HLA allele (*h*_1+_; see the “Methods” section). The AUC for *h*_1+_ was 0.98 for MHC class I alleles and 0.81 for MHC class II alleles (Additional file 1: Fig. S18). Germline/host HLA homozygosity has been posited as a biomarker of response to immunotherapy that is independent of somatic HLA alterations [48, 51], underscoring the importance of accurate germline imputation (our focus here). Overall, tumor imputed variants can thus be directly used for downstream imputation of HLA alleles at a similar level of accuracy.

### Inference of germline polygenic risk scores

**Fig. 4:**
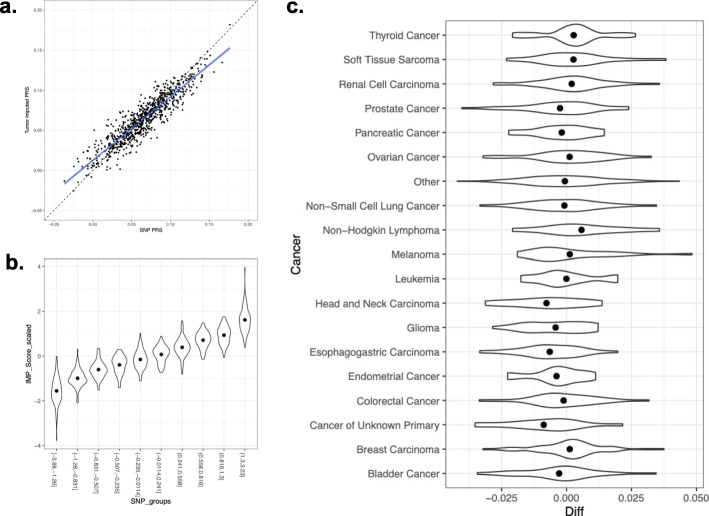
Polygenic risk score (PRS) accuracy. **a** Scatter plot of germline SNP (*x*-axis) and tumor imputed (*y*-axis) polygenic risk score across individuals. Each dot is an individual; dashed line shows *y* = *x* diagonal; blue line shows the linear fit. **b** Violin plot of imputed score density (*y*-axis) as a function of genotyped score decile (*x*-axis). **c** Violin plot of the density of imputed/genotyped score differences (*x*-axis) across cancer types (*y*-axis)

Common germline variants are increasingly being used to predict disease risk by aggregating individual effect sizes into polygenic risk scores (PRSs) [52, 53] and we investigated the accuracy of PRSs computed from the tumor-imputed variants. We selected the PRS from a recent large-scale breast cancer GWAS [38] as representative (findings were similar for other PRSs from polygenic traits: Additional file 1: Fig. S19). For each individual, a risk score was computed using the tumor imputed variants and compared to that computed using the germline genotypes. Pearson correlation across individuals for the two scores was 0.92 with no observable outliers, and a slight linear deflation of the score as would be expected from noise due to imputation (Fig. 4a). We confirmed that PRS imputation error (computed as the difference between the imputed and true PRS) was approximately normally distributed (Additional file 1: Fig. S20) and consistent across the true PRS deciles (Fig. 4b). Lastly, we found no statistically significant difference in the mean error across cancer types (in a multivariate linear regression; Fig. 4c), nor was any cancer type individually associated with mean error or mean absolute error. Likewise, no significant biases in the mean PRS error were observed across the sequencing panel versions (Additional file 1: Fig. S21), although the variance in error differed, as expected from differing levels of imputation noise across panels (see above). Other common PRS phenotypes yielded similar performance (Additional file 1: Fig. S19).

**Fig. 5:**
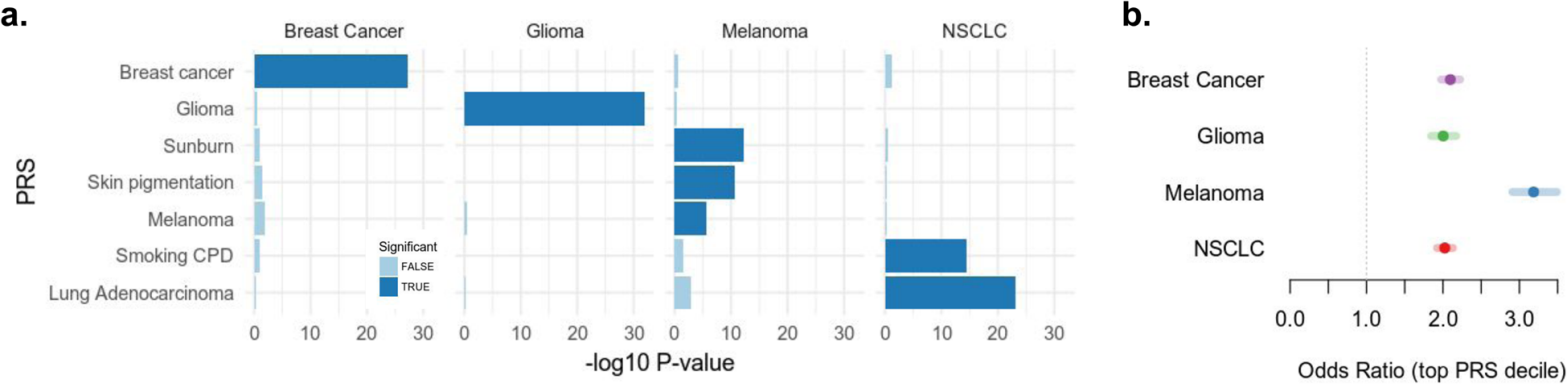
PRS associations with cancer types. **a** Each row corresponds to a different tested PRS, and each column shows a different tumor/cancer type. Bars (*x*-axis) show the significance of the PRS-cancer type association on the -log_10_ *p*-value scale (higher = more significant). Bonferroni significant associations are shown in darker shades. Note that the significance of the PRS depends on both prediction accuracy as well as target sample size and should not be directly compared across cancer types. **b** Odds ratio (*x*-axis) of the top risk PRS decile associated with the corresponding cancer type shown for each cancer type (*y*-axis). The shaded bar shows the standard error of the estimate from a logistic regression

Having established the high accuracy of the tumor imputed PRS, we applied it to the full cohort of tumors to investigate the differences between cancers. We constructed PRSs for common cancers (breast, glioma, lung, and melanoma) as well as risk PRSs for exposures typically associated with these cancers (smoking and tanning), using genome-wide significant variants from corresponding GWAS (see the “Methods” section). We note that a PRS for an exposure is a prediction of the genetic predisposition for the corresponding behavior (e.g., propensity to smoke) and not a direct measurement of the exposure. We then tested each score for association with cancer type, with cases defined as patients having a focal cancer and controls defined as patients with any other cancer (note, no genuine healthy controls were available in this cancer cohort). Each risk PRS was highly significantly associated with tumors of the respective cancer type (Fig. 5a), serving as a validation of both the scores and the imputed variants. Additionally, the smoking PRS was associated with lung tumors, and the pigment/sunburn PRS was associated with melanoma tumors as anticipated. No significant associations were observed for any mismatching PRS/cancer pairs, confirming empirically that our approach did not induce cancer-specific biases. Testing individuals in the top risk score decile yielded odds ratios of 2–3 (Fig. 5b), which has been posited as the range for potential clinical actionability [52]. In sum, this analysis demonstrates sufficient accuracy of tumor-imputed PRSs to explore risk/exposure relationships and identify individuals at the risk score extremes.

### Inference of genetic ancestry

**Fig. 6:**
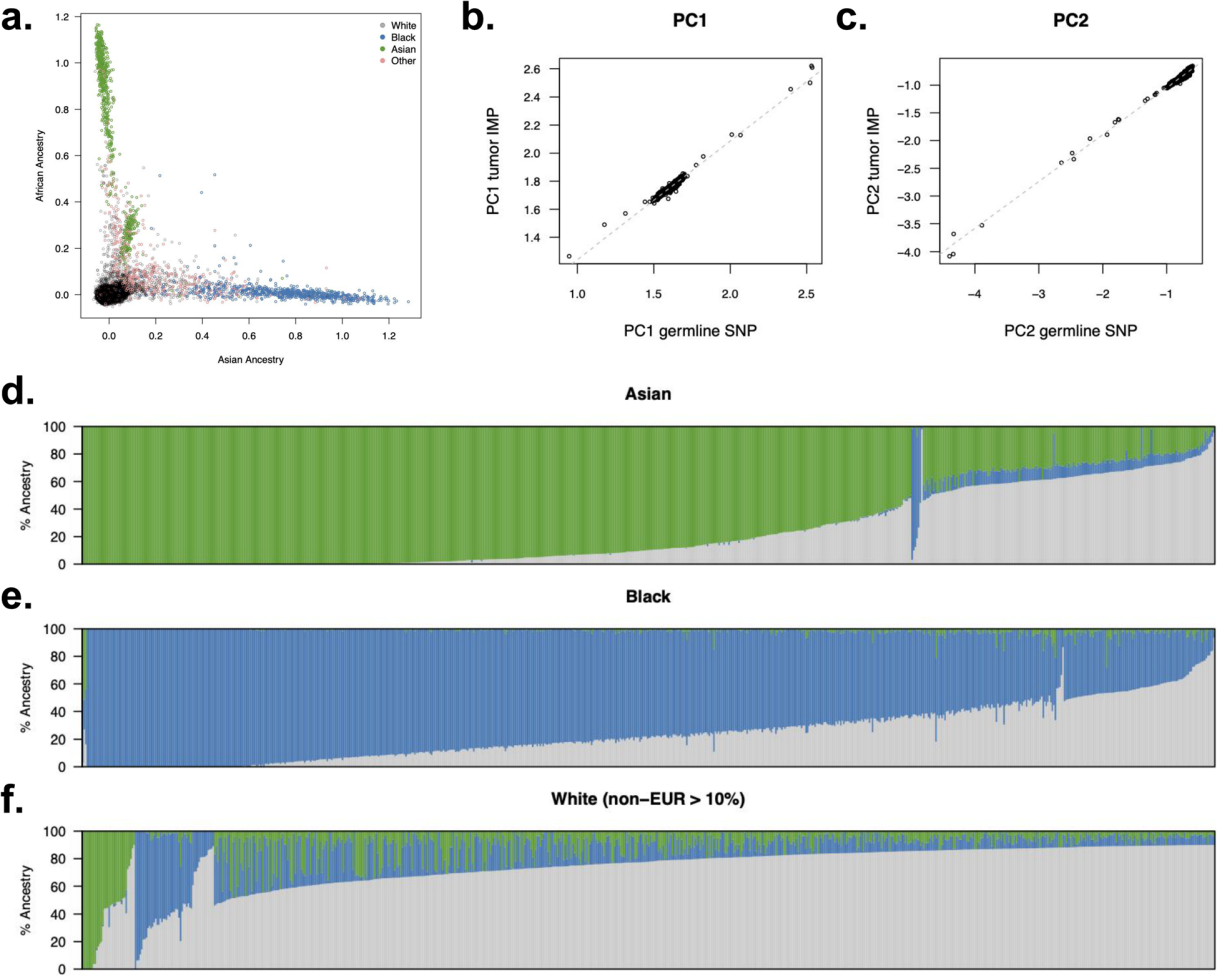
Genetic ancestry inferred from tumor sequencing. **a** Projected continental ancestry components for all individuals. Each dot is a projected sample (color-coded by self-reported race); the *x*-axis is the Asian ancestry component, and the *y*-axis is the African ancestry component. **b**, **c** Correlation of imputed/genotyped ancestry components 1 and 2, respectively. **d**–**f** Ancestry fractions for individuals self-reporting as Asian, Black, and White (with > 10% inferred non-European ancestry)

Germline variants can be used to discover and estimate genetic ancestry components, which are often partially distinct from self-reported race and ethnicity [54, 55]. Previous work has shown that genetic data from an individual of unknown ancestry can be “projected” onto their ancestry component using weights from a reference panel [45] (akin to an ancestry PRS). We investigated this approach to ancestry inference by projecting tumor imputed genotypes into the principal components of three continental populations (European, African, and Asian; inferred from the 1000 Genomes Project reference data). The inferred ancestry revealed clines consistent with self-reported race (Fig. 6a). Using the benchmarking samples, the correlation of ancestry estimates from tumor imputed variants versus germline genotyped variants was > 0.98 with no significant outliers (Fig. 6b, c). Continental ancestry was thus inferred from tumor imputed data with nearly perfect accuracy. We note that prior work showed ancestry projections from reference data are more accurate than in-sample principal component analysis [45], and thus, we did not investigate the latter in the tumor data.

Having established accurate inference of genetic ancestry, we examined the variance in genetic ancestry across our full cohort of ~ 25,000 samples. We first focused on individuals self-reporting as Asian (*n* = 687) or Black (*n* = 750) in the electronic health record (Fig. 6d,e). As expected, self-reported Asian and Black patients exhibited predominantly East Asian and West African ancestry, respectively. However, admixture from other populations was also present in these samples: 50% of self-reported Asian patients had > 10% ancestry from a non-Asian population, and 72% of self-reported Black patients had > 10% ancestry from a non-African population. Next, we turned to self-reported White individuals with > 10% non-European ancestry (*n* = 730 out of 21,451 total self-reported Whites; Fig. 6f). In addition to observing *n* = 25 individuals with > 50% East Asian ancestry and *n* = 33 individuals with > 50% African ancestry (possibly reflecting miscoded EHR data), we observed a cline of individuals with ancestry from both populations, likely encompassing Hispanic/Latino individuals who often exhibit this pattern of admixture [56] and self-report as White.

To demonstrate the relationship between genetic ancestry and somatic features, we focused on 2900 non-small cell lung cancer (NSCLC) patients with somatic, non-synonymous SNVs in the *EGFR* gene, which are known to have higher frequencies in Asians [57, 58]. Restricting to individuals with Asian or European ancestry, we confirmed a highly significant increase in *EGFR* mutation rate in tumors from individuals with Asian ancestry (18% in samples of European ancestry; 57% in samples of Asian ancestry; *p* = 3 × 10^−22^ by logistic regression). Genetic ancestry was more significantly associated with *EGFR* status than self-reported race (*p* = 1.0 × 10^−3^ for ancestry and *p* = 0.33 for race, in a joint model; Additional file 1: Table S3a), highlighting the utility of the additional variation derived from inferred ancestry. Indeed, individuals that were self-reported White but had East Asian ancestry carried somatic *EGFR* SNVs at nearly the same rate as self-reported Asian individuals (47% and 58%, respectively; Additional file 1: Table S3b), serving as independent support of the inferred ancestry in these race/ancestry discordant samples.

## Discussion

Traditionally, research cohorts for identifying germline and somatic cancer factors have been examined with different technologies and analysis methods, each optimized to specific goals. GWAS have identified over 1000 SNPs associated with susceptibility to over 30 cancer types [59, 60], but current GWAS techniques do not examine tumor sequences. Cancer-oriented cohorts like TCGA [61] obtained multiple functional data types, but sample sizes were too small for GWAS analyses, clinical data were sparse [62], and the patient populations had limited longitudinal follow-up. New multi-consortium studies aim to increase the sample size, but the high cost of whole-genome sequencing remains a limiting factor [16]. Targeted panel sequencing of tumors thus offers a reduced cost, and the rich clinical data collected across hundreds of thousands of patients [11] provide an unprecedented opportunity for basic research and translational discovery. However, the lack of genome-wide germline data for these studies has limited the capacity to integrate GWAS-like analyses with somatic outcomes.

To overcome this gap between germline and somatic studies, we implemented and validated an imputation framework to derive germline genotypes and downstream features directly from targeted tumor sequencing. Our pipeline offers the opportunity to run germline GWAS, estimate polygenic risk for complex phenotypes, and assign genetic ancestry. We demonstrated feasibility in real-world data from > 25,000 tumors, identifying highly significant PRS associations and novel ancestry diversity. For individuals in the highest PRS decile of cancer risk, we observed corresponding odds ratios of > 2, reaching the range of potential individual-level actionability. Ancestry scores, on the other hand, were imputed with near-perfect accuracy, providing a framework to easily incorporate genetic ancestry into the study of tumors from existing large-scale datasets and expand our knowledge of population-specific mechanisms [63]. Multiple emerging studies have demonstrated the utility of low-coverage sequencing of normal samples [18, 22, 23], and our work extends these findings to targeted, tumor-only data.

Our approach has several important limitations. First, while we show that ancestry and polygenic risk can be imputed with high and moderate-to-high accuracy, respectively, and negligible bias; individual variant calls should always be evaluated in the context of their imputation uncertainty. We observed a mean imputation correlation of 0.86 which, under standard assumptions of linearity, is expected to translate into an effective sample size of 0.86^2^ = 0.74× relative to direct germline genotyping [17]. This approach is therefore most applicable to GWAS discovery, and individual imputed alleles must be interpreted with care. Second, while most genotyping errors will result in decreased sensitivity that may be acceptable in a discovery analysis, some forms of germline-somatic analysis may produce false-positive associations. For example, we found that deep somatic deletions introduced noise and shifted the mean imputed variant toward the reference/common allele. This allele frequency shift may appear as a false-positive association between the germline variant and a recurrent deletion (or other SCNAs correlated with the deletion). When testing individual variants in loss-prone regions, we therefore recommend either excluding individuals that carry a local deletion, or meta-analyzing carriers and non-carriers. Third, our study was limited to working with a single tumor-only panel sequencing platform (OncoPanel) which may not generalize to platforms used at other institutions. However, we evaluated four versions of this panel (which differed in their gene targets, sequencing chemistry, and years of use) and observed the mean imputation accuracy in the range of 0.79–0.88 across panels and highly consistent across the genome. Moreover, > 80% of the variance in individual imputation accuracy was explained by panel version and off-target coverage, suggesting that the performance we observed can be extrapolated to other panels, though broader evaluation is needed. For studies that perform tumor/normal matched sequencing, we expect that imputation from the normal sample would only further increase the accuracy due to the lack of confounding from somatic alterations (consistent with our observation that tumor purity slightly increased imputation error even in this tumor-only cohort). However, the optimal combination of tumor/normal data and sequencing platform for germline imputation remains an open question.

The availability of germline genotypes in somatically phenotyped, clinical cohorts offers multiple new research opportunities. Studies of germline-somatic interactions have been held back by the need to collect both modalities in the same individuals, which has limited the scope of such analyses to merely hundreds of patients per cancer [64]. The availability of germline variation in hundreds of thousands of tumors can enable GWAS-scale studies of somatic alterations and broaden our understanding of tumor evolution. Even merely using existing tumor sequencing to increase the number of cases available for conventional GWAS would be meaningful; for example, AACR Project Genie has sequenced ~ 17,000 lung and ~ 6700 glioma tumors, which would substantially expand the ~ 30,000 lung cases and ~ 12,500 glioma cases in the largest corresponding GWAS studies that exist to date [39, 40]. Lastly, and perhaps most importantly, as tumor sequencing is part of routine clinical care at many institutions, off-target imputation could be directly incorporated into existing workflows and greatly accelerate the translation of germline biomarkers and PRSs to the clinic. To facilitate this research, code for all analyses described here is available in a repository and deployable pipeline (see the “Availability of data and materials” section).

## Conclusions

In conclusion, we show that common germline genotypes and derivative features can be accurately imputed from tumor-only panel sequencing. Our framework is publicly available and lays the path for germline studies from hundreds of thousands of tumors in existing datasets.

## Supplementary Information

**Additional file 1: Figure S1.** Histogram of broad cancer types in the full tumor cohort. **Figure S2.** Coverage histogram. **Figure S3.** Imputation accuracy by filtering criteria. **Figure S4.** Robustness of imputation correlation estimate. **Figure S5.** Copy neutral loss of heterozygosity calling (CN-LOH). **Figure S6.** Distribution of imputation correlation across all (pre-filtered) HapMap3 variants by imputation scheme (x-axis and color code). **Figure S7.** Distribution of imputation correlation by INFO score and coverage. **Figure S8.** Cumulative imputation accuracy. **Figure S9.** Imputation correlation by variant type. Figure S10. Imputation correlation for pseudo-SNP indels. **Figure S11.** Manhattan plot of imputation correlation across panel versions. **Figure S12.** Distribution of imputation allelic error across sequencing panels. **Figure S13.** Distribution of imputation allelic error by coverage and panel. **Figure S14.** Variance in imputation error explained by technical features. **Figure S15.** Imputation error by tumor TMB and FFPE sample. **Figure S16.** Imputation error by tumor purity. **Figure S17.** Percent of SNPs with high levels of error at somatically altered regions. **Figure S18.** HLA homozygosity calling accuracy. **Figure S19.** PRS imputation accuracy. **Figure S20.** Breast PRS error. **Figure S21.** PRS mean error by panel. **Table S1.** Association of somatic features with imputation error. **Table S2.** Number of somatic SNVs per sample that overlap a common reference panel variant in PCAWG tumor WGS data. **Table S3.** EGFR associations with race and ancestry.

## Data Availability

All unidentifiable data is available within the article and its supporting files. The individual-level sequencing and imputation data cannot be made publicly available because the research participant consent does not include authorization to share identifiable data. The full analysis workflow is available at https://github.com/gusevlab/panel-imp [65]. A containerized version of the imputation pipeline is available at https://hub.docker.com/r/stefangroha/stitch_gcs.
