# Supplementary material for: Constructing germline research cohorts from the discarded reads of clinical tumor sequences
Source: Genome Med. 2021 Nov 8;13:179. doi: 10.1186/s13073-021-00999-4 (PMC8576948; doi:10.1186/s13073-021-00999-4)
Supplement: Supplementary file 1 — Additional file 1: Figure S1. Histogram of broad cancer types in the full tumor cohort. Figure S2. Coverage histogram. Figure S3. Imputation accuracy by filtering criteria. Figure S4. Robustness of imputation correlation estimate. Figure S5. Copy neutral loss of heterozygosity calling (CN-LOH). Figure S6. Distribution of imputation correlation across all (pre-filtered) HapMap3 variants by imputation scheme (x-axis and color code). Figure S7. Distribution of imputation correlation by INFO score and coverage. Figure S8. Cumulative imputation accuracy. Figure S9. Imputation correlation by variant type. Figure S10. Imputation correlation for pseudo-SNP indels. Figure S11. Manhattan plot of imputation correlation across panel versions. Figure S12. Distribution of imputation allelic error across sequencing panels. Figure S13. Distribution of imputation allelic error by coverage and panel. Figure S14. Variance in imputation error explained by technical features. Figure S15. Imputation error by tumor TMB and FFPE sample. Figure S16. Imputation error by tumor purity. Figure S17. Percent of SNPs with high levels of error at somatically altered regions. Figure S18. HLA homozygosity calling accuracy. Figure S19. PRS imputation accuracy. Figure S20. Breast PRS error. Figure S21. PRS mean error by panel. Table S1. Association of somatic features with imputation error. Table S2. Number of somatic SNVs per sample that overlap a common reference panel variant in PCAWG tumor WGS data. Table S3. EGFR associations with race and ancestry. [file 13073_2021_999_MOESM1_ESM.pdf]

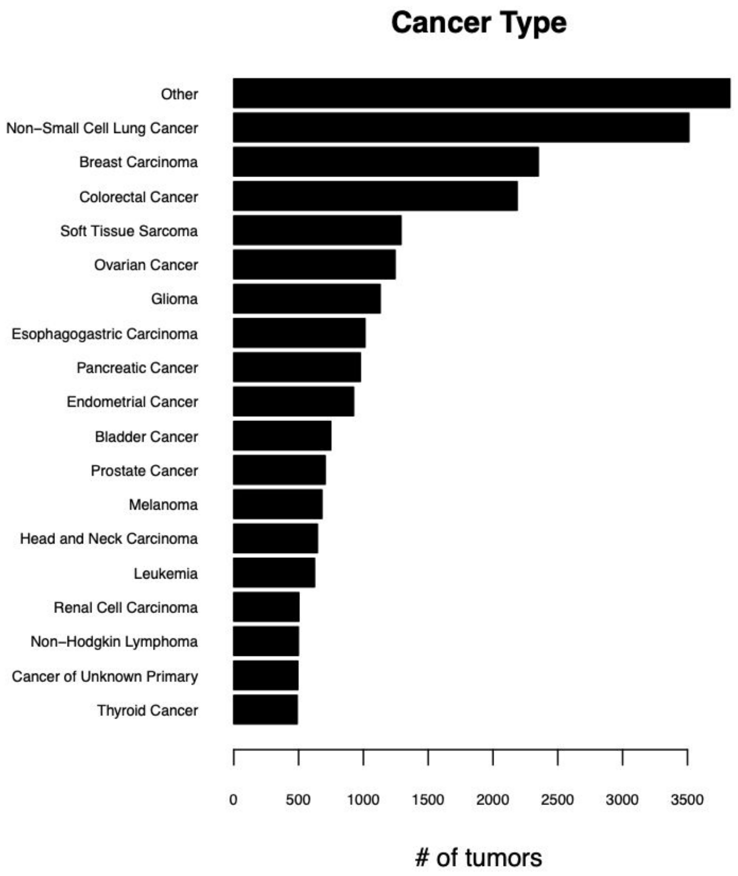

Fig S1: Histogram of broad cancer types in the full tumor cohort.

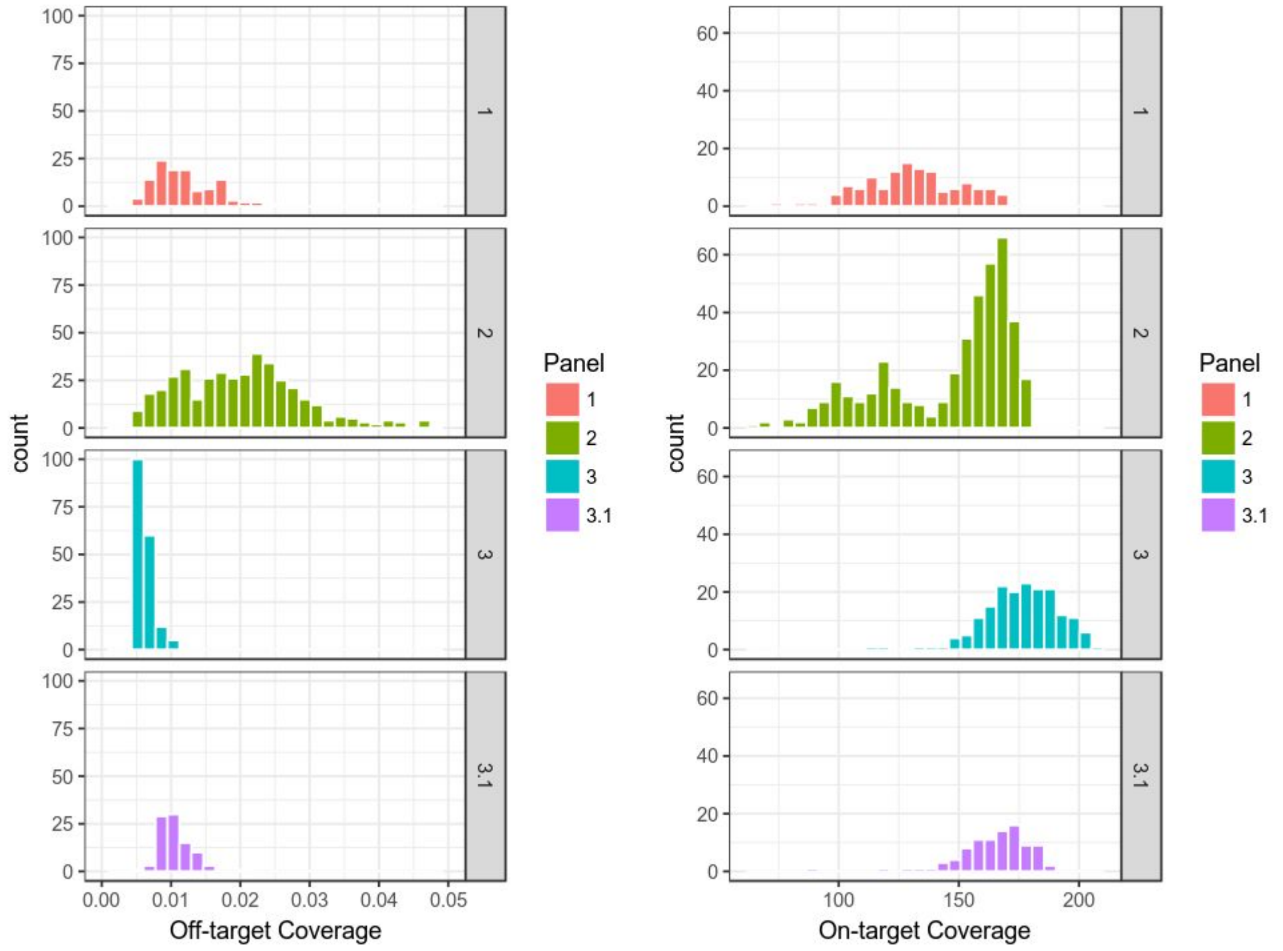

Fig S2: Coverage histogram. Histogram of off-target coverage (left) and on-target coverage (right) by panel version (vertical facets and colors).

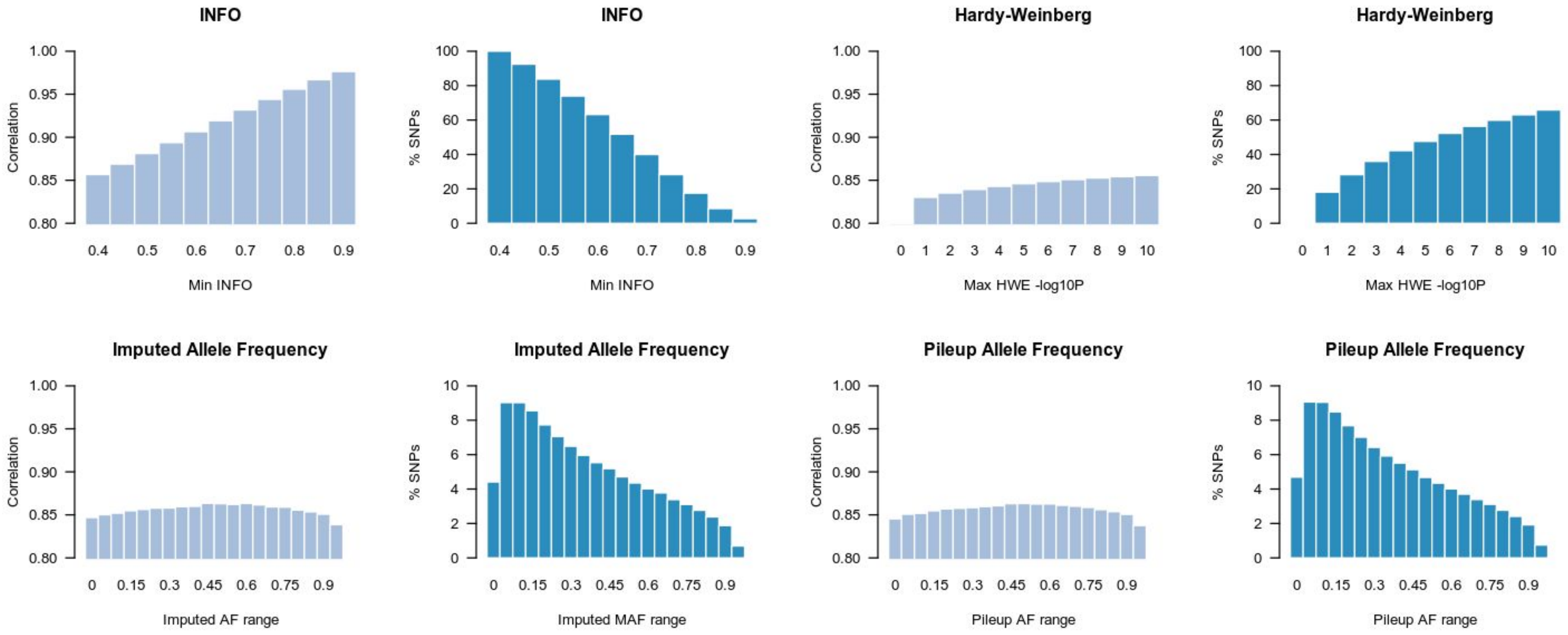

Fig S3: Imputation accuracy by filtering criteria. Histogram of imputation correlation (light blue) and fraction of SNPs (dark blue) as a function of filtering thresholds. INFO: Imputation confidence score; HWE: Hardy-Weinberg equilibrium p-value; EAF: estimated allele frequency; PAF: estimated allele frequency based on reads.

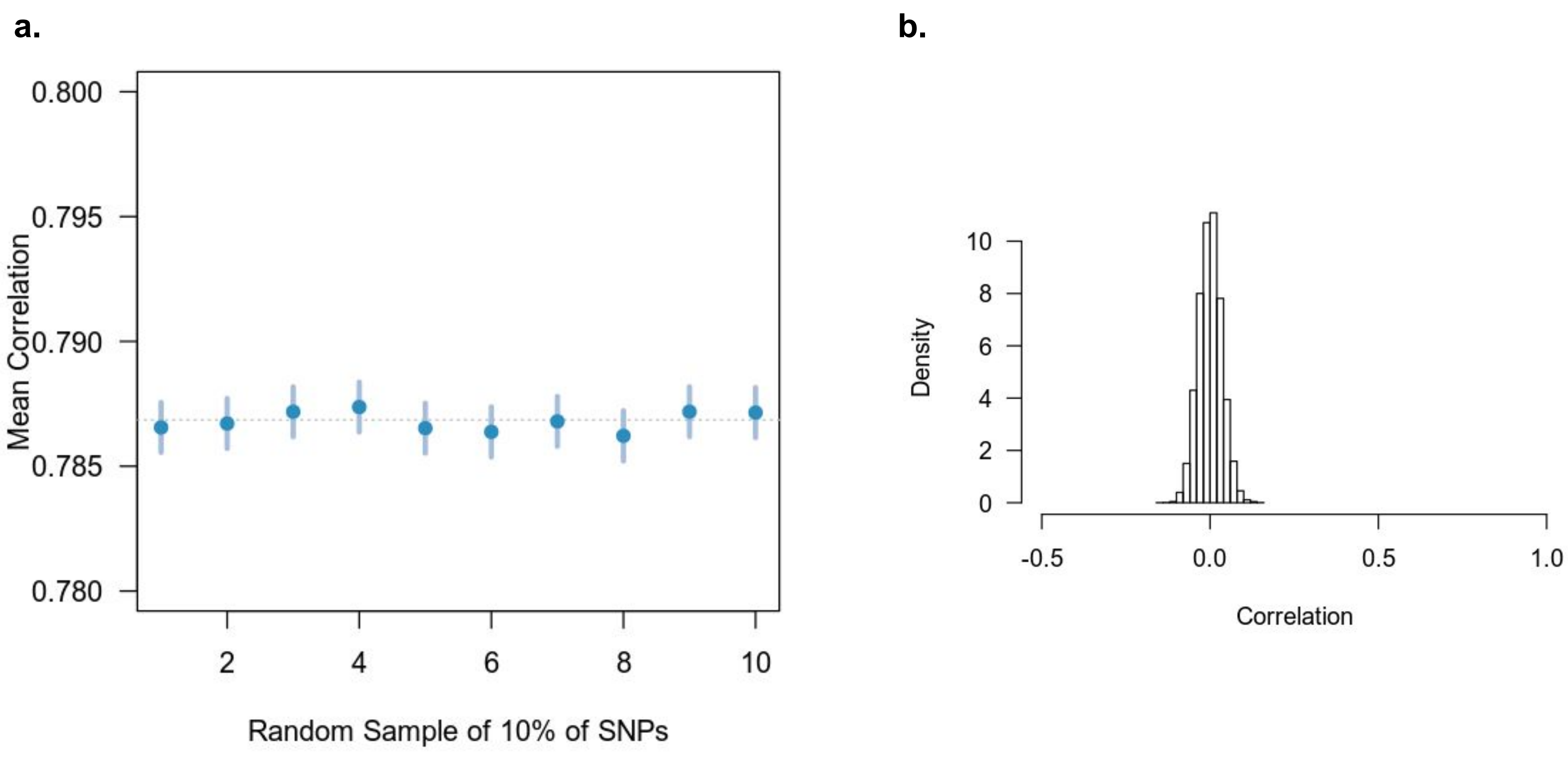

Fig S4: Robustness of imputation correlation estimate. (**a**) Mean imputation correlation (y-axis) estimated across ten random down-sampling to 10% of SNPs. (**b**) Distribution of mean Imputation correlation across 100 sample permutations, where sample labels were shuffled in each permutation.

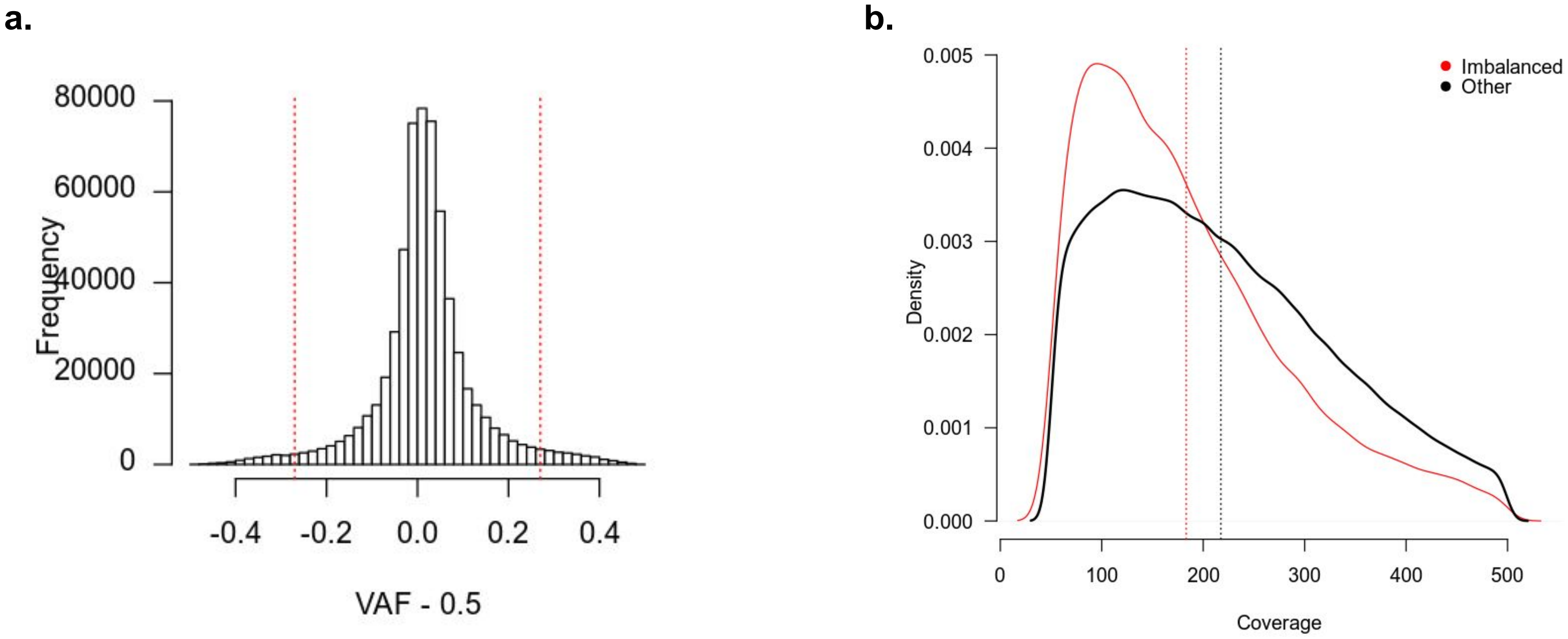

Fig S5: Copy neutral loss of heterozygosity calling (CN-LOH). (**a**) Histogram of variant allele frequency (VAF) deviation from somatic reads at germline heterozygous variants, defined as the VAF - 0.5 (x-axis). Vertical red lines indicate thresholds that were used to define imbalanced sites for putative CN-LOH. (**b**) Coverage distribution for imbalanced sites with putative CN-LOH (above/below the threshold in (**a**)) versus other on-target sites.

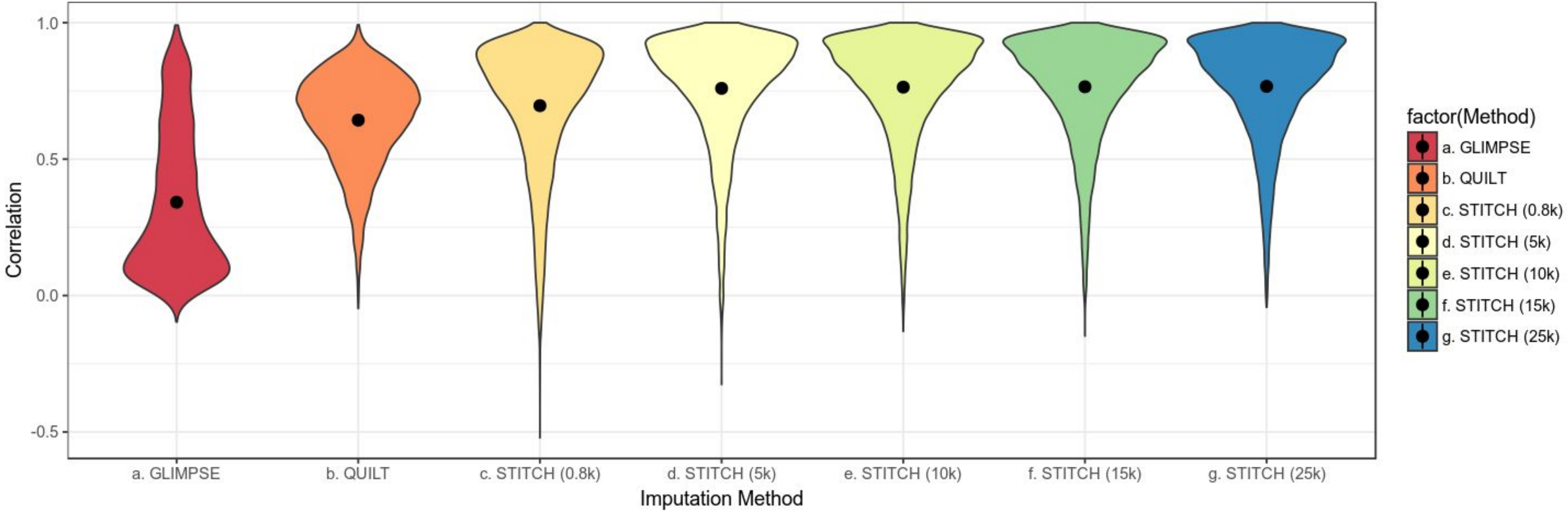

Fig S6: Distribution of imputation correlation across all (pre-filtered) HapMap3 variants by imputation scheme (x-axis and color code).

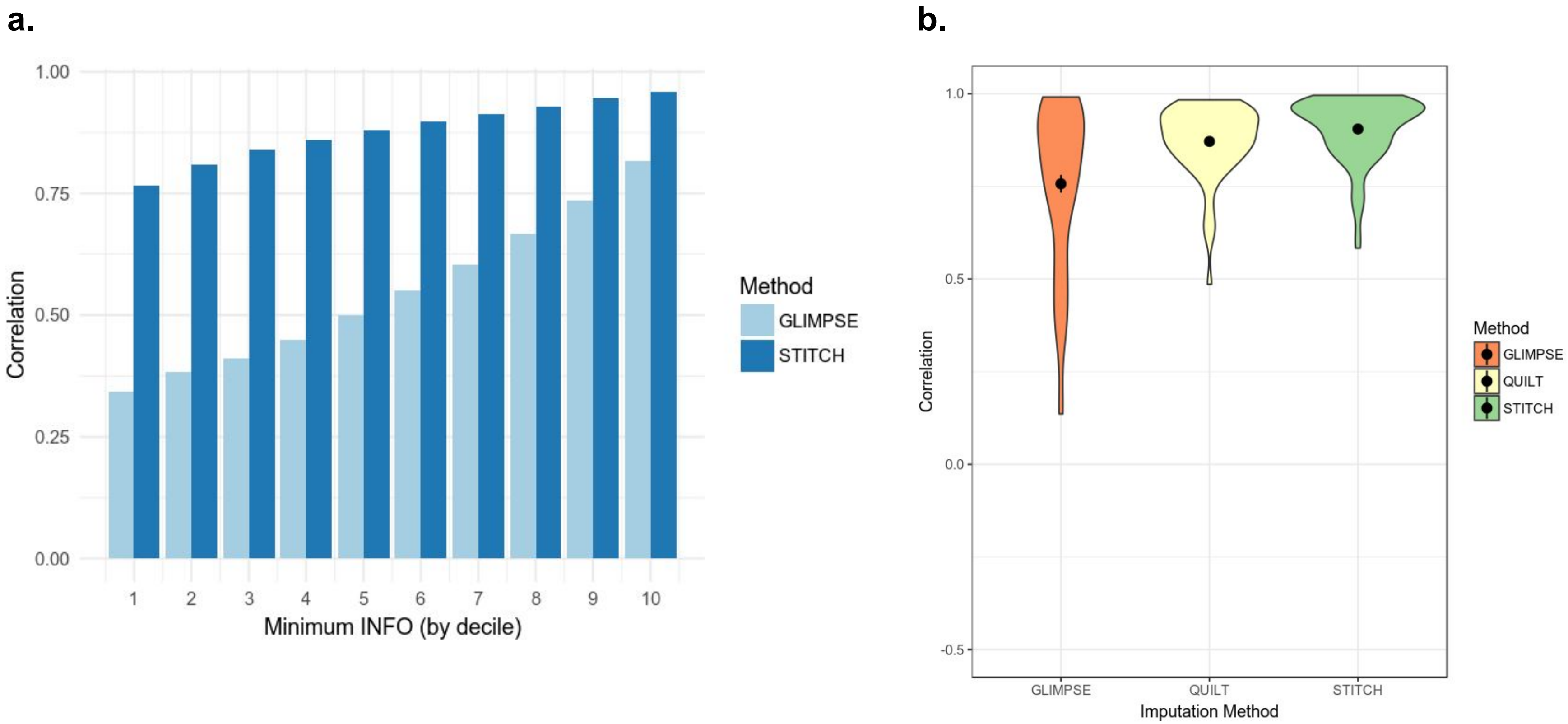

Fig S7: Distribution of imputation correlation by INFO score and coverage. (**a**) Distribution of imputation correlation (y-axis) by INFO score decile (x-axis) and imputation algorithm (GLIMPSE: light blue; STITCH: dark blue). (**b**) Distribution of imputation correlation for high-coverage variants (mean coverage >10x) across three methods.

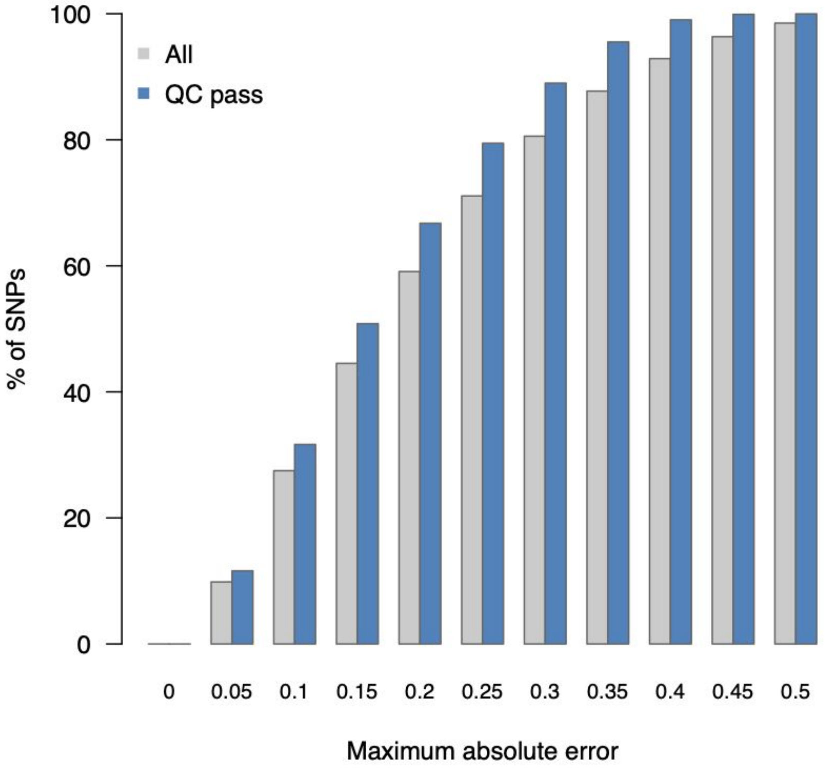

Fig S8: Cumulative imputation accuracy. Histogram of fraction of SNPs (y-axis) as a function of maximum absolute imputation error (x-axis) for all imputed SNPs (gray) and filtered SNPs (INFO>0.4, MAF>1%) (blue).

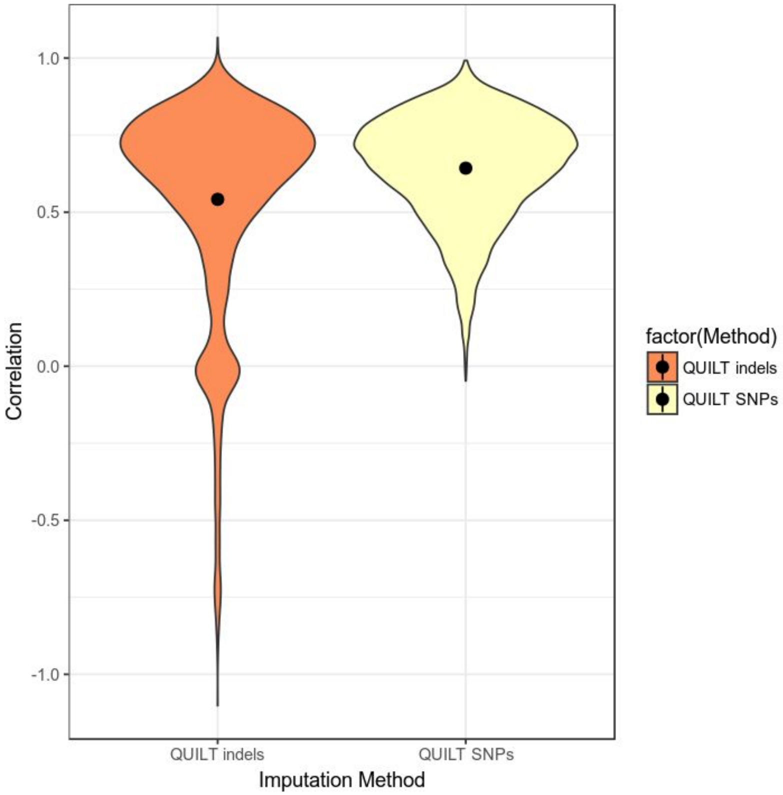

Fig S9: Imputation correlation by variant type. Distribution of imputation correlation (y-axis) from QUILT by variant type (x-axis and color; orange: indels, yellow: SNPs).

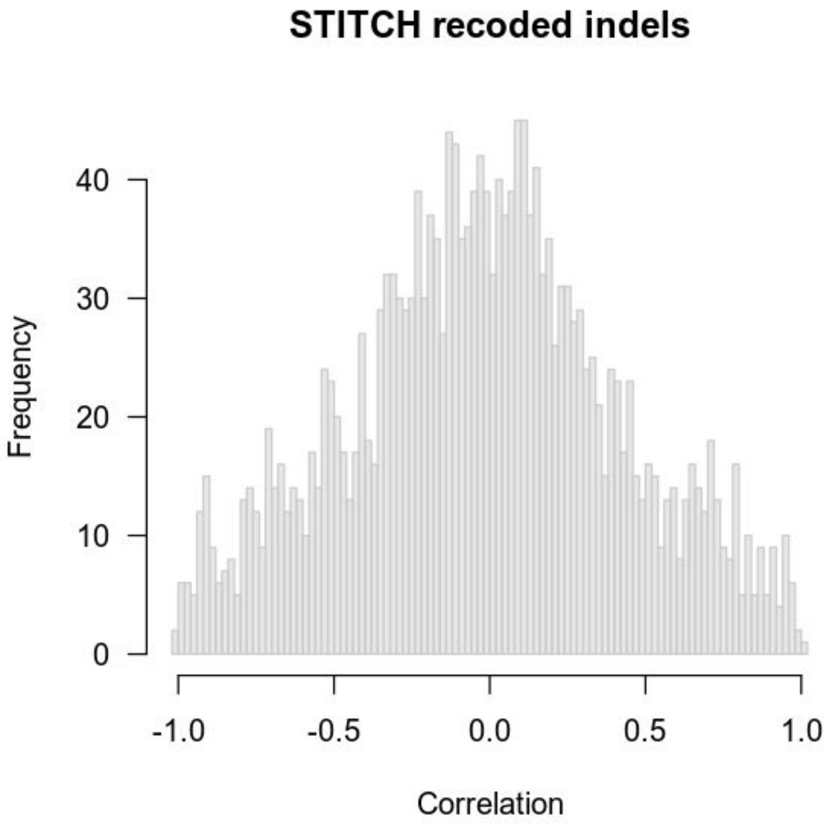

Fig S10: Imputation correlation for pseudo-SNP indels. Histogram of imputation correlation (x-axis) for indels using STITCH imputation after recoding indels as bi-allelic “pseudo-SNPs”. This re-encoding likely leads to improper interpretation of low-coverage reads overlapping true indels and yields low indel imputation accuracy.

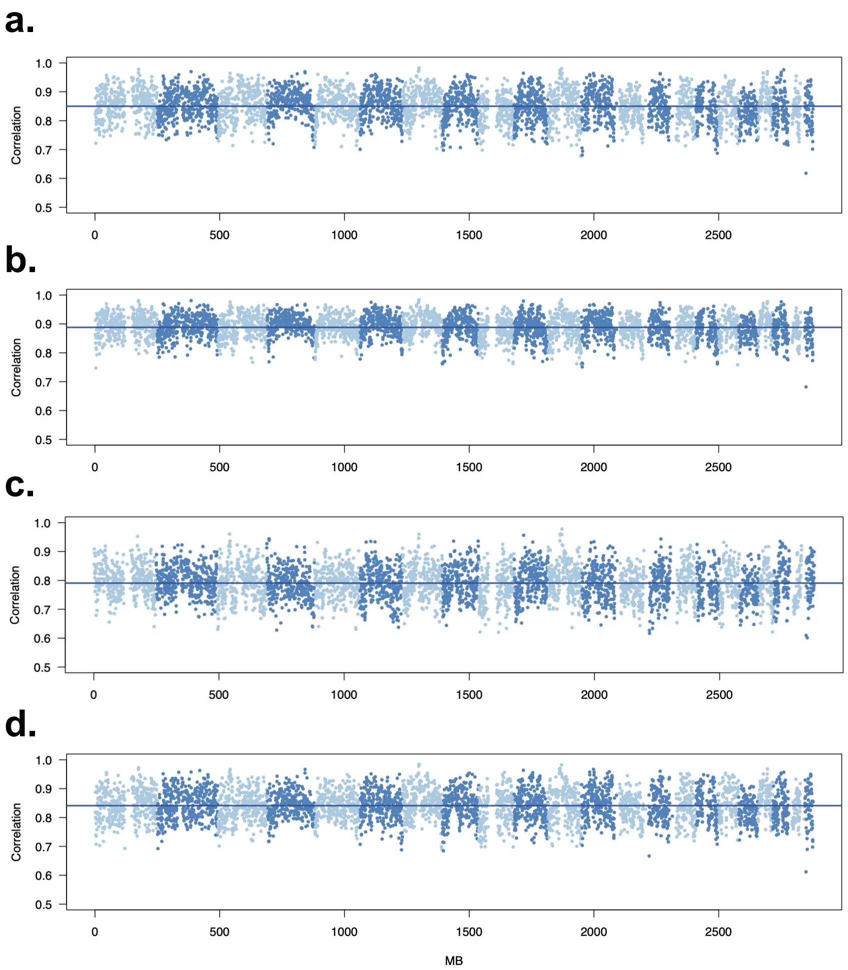

Fig S11: Manhattan plot of imputation correlation across panel versions. (a) v1, (b) v2, (c) v3, (d) v3.1. Alternating colors represent chromosomes.

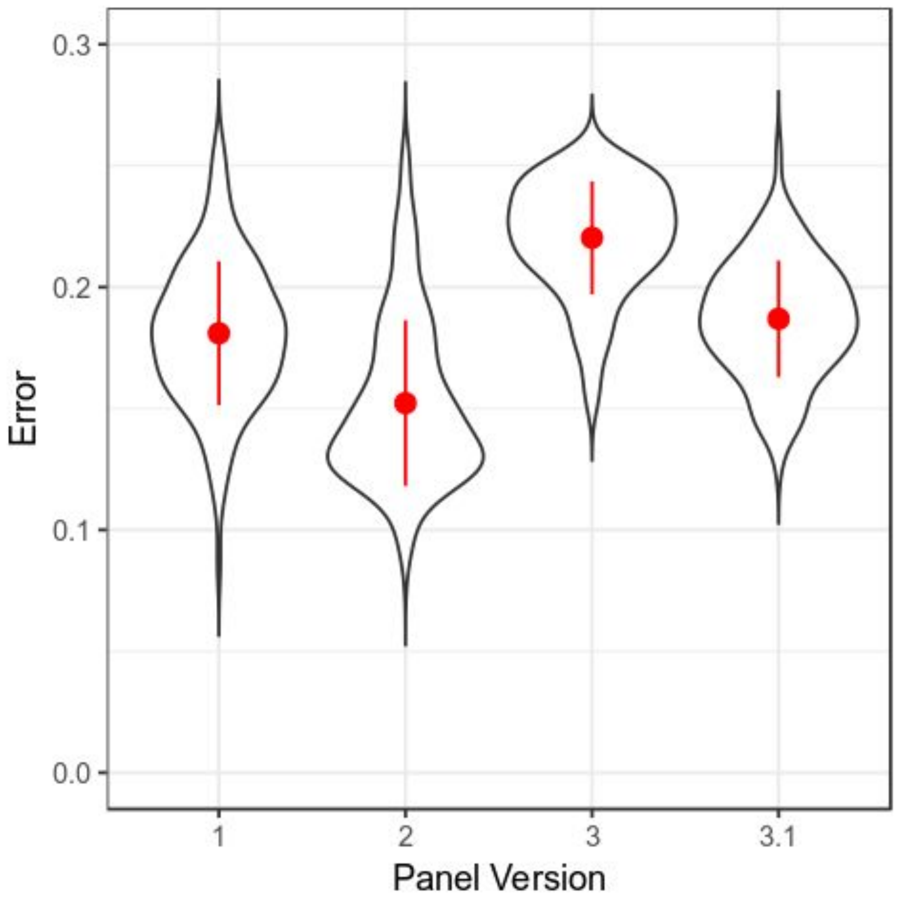

Fig S12: Distribution of imputation allelic error across sequencing panels. Lower means lower error / higher quality. Red point indicates mean and red line indicates standard deviation.

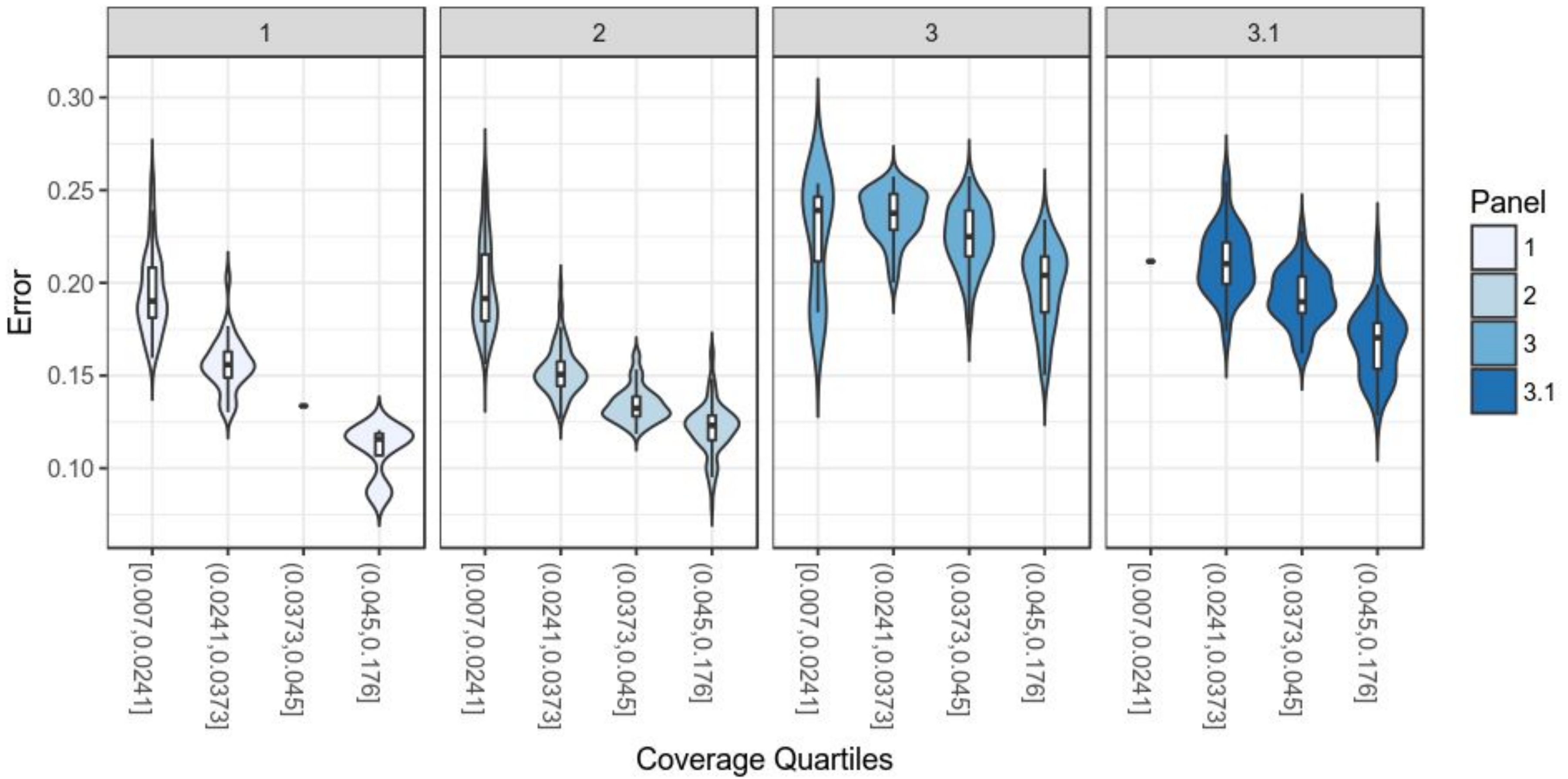

Fig S13: Distribution of imputation allelic error by coverage and panel. Distribution of imputation error (y-axis) by coverage quartile (x-axis) and panel version (facets and colors). Lower means lower error / higher quality.

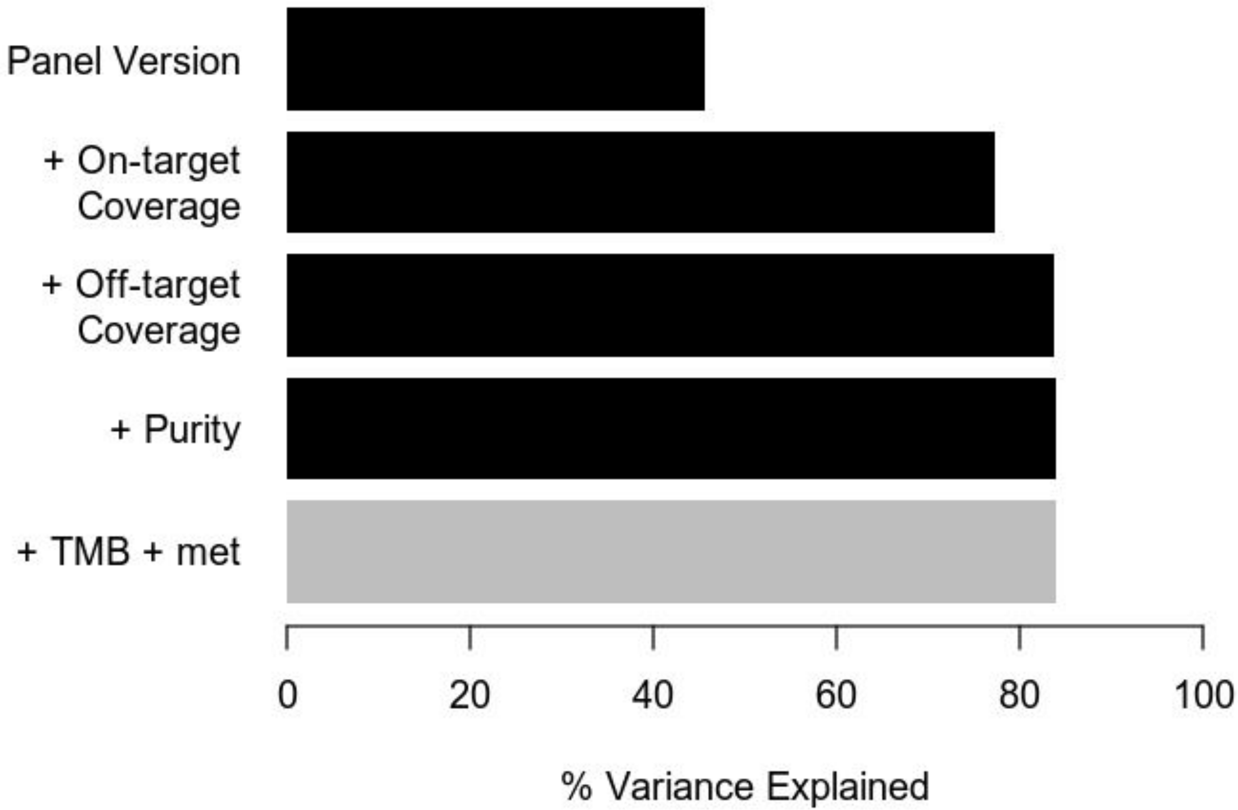

Fig S14: Variance in imputation error explained by technical features. Individual-level variance in mean imputation error (x-axis) explained by various models of technical features (y-axis). From top to bottom models are nested and include all previous features).

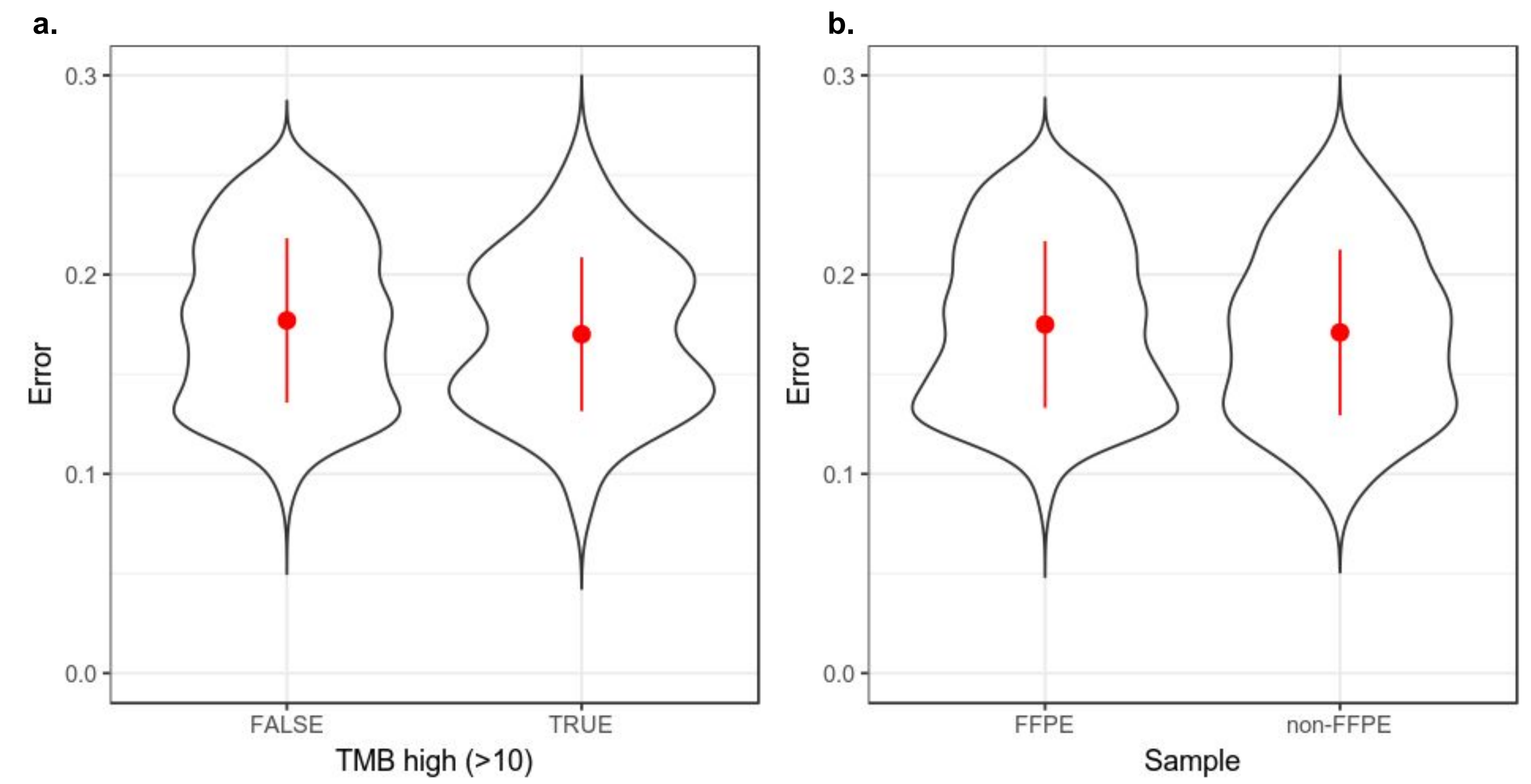

Fig S15: Imputation error by tumor TMB and FFPE sample. Distribution of imputation allelic error (y-axis) for individuals with (**a**) low/high TMB (>10 mutations/MB; x-axis) and (**b**) FFPE vs non-FFPE (n=108) tissue source. Red point indicates mean and red line indicates standard deviation.

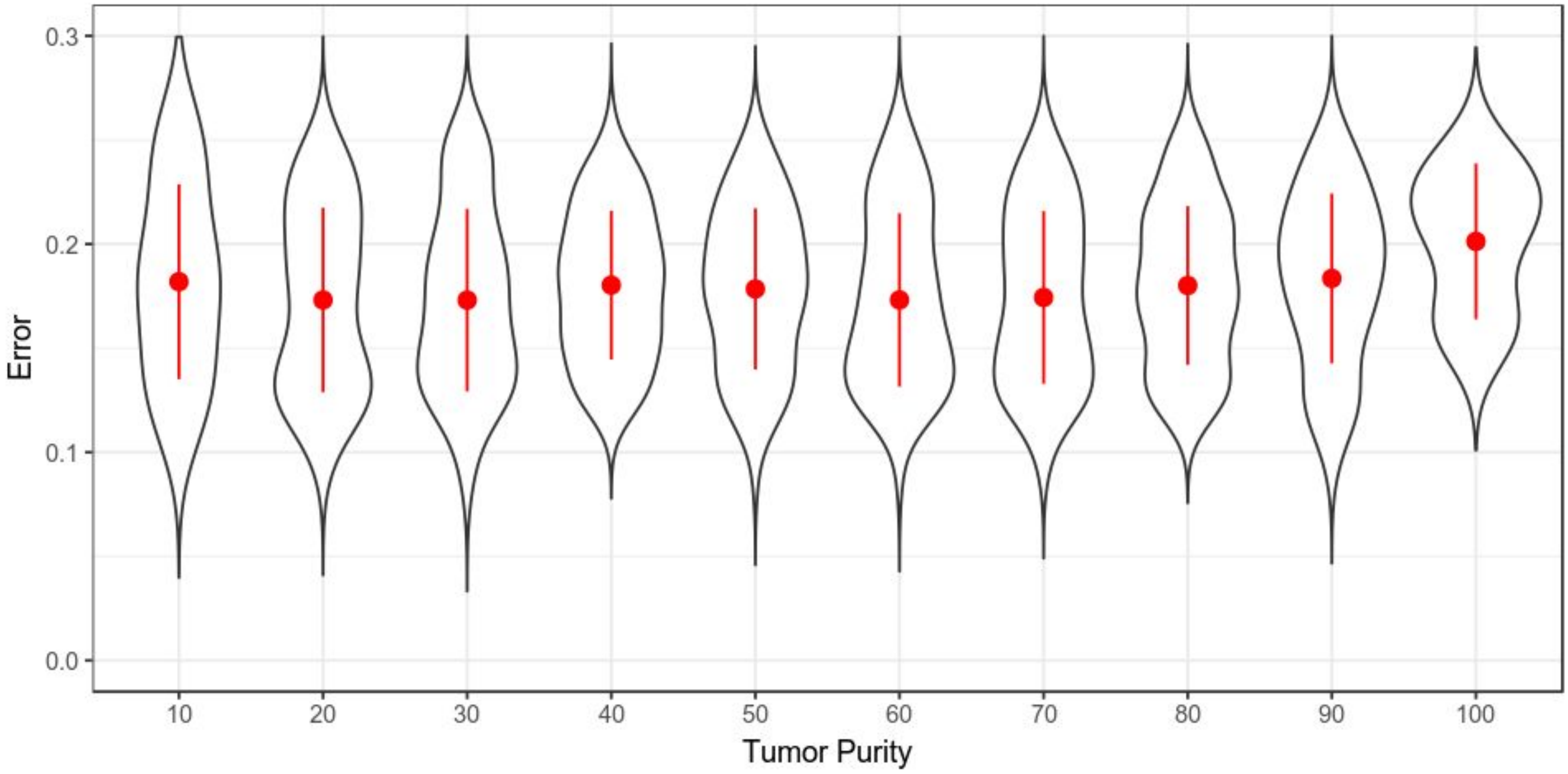

Fig S16: Imputation error by tumor purity. Distribution of imputation allelic error (y-axis) as a function of tumor purity (x-axis). Red point indicates mean and red line indicates standard deviation.

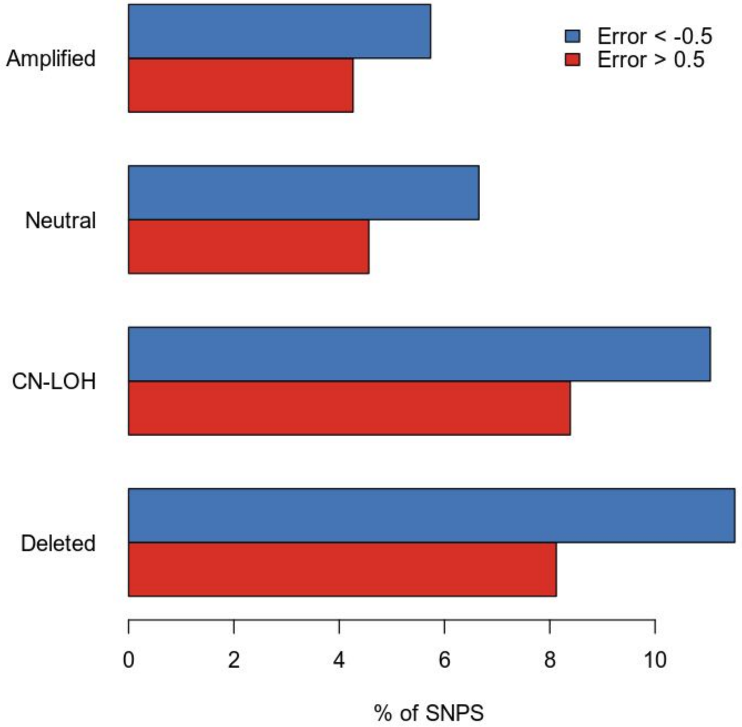

Fig S17: Percent of SNPs with high levels of error at somatically altered regions. Each bar shows the fraction of SNPs within the named region type having an allelic error of <-0.5 (blue, bias towards the major allele) or >0.5 (red, bias towards the minor allele). From top to bottom, regions correspond to: 5% most amplified, neutral (not covered by any other category), 5% most copy-neutral loss of heterozygous (CN-LOH), 5% most deleted. All regions exhibit a slight bias towards the major allele, with CN-LOH and Deleted regions exhibiting greater bias.

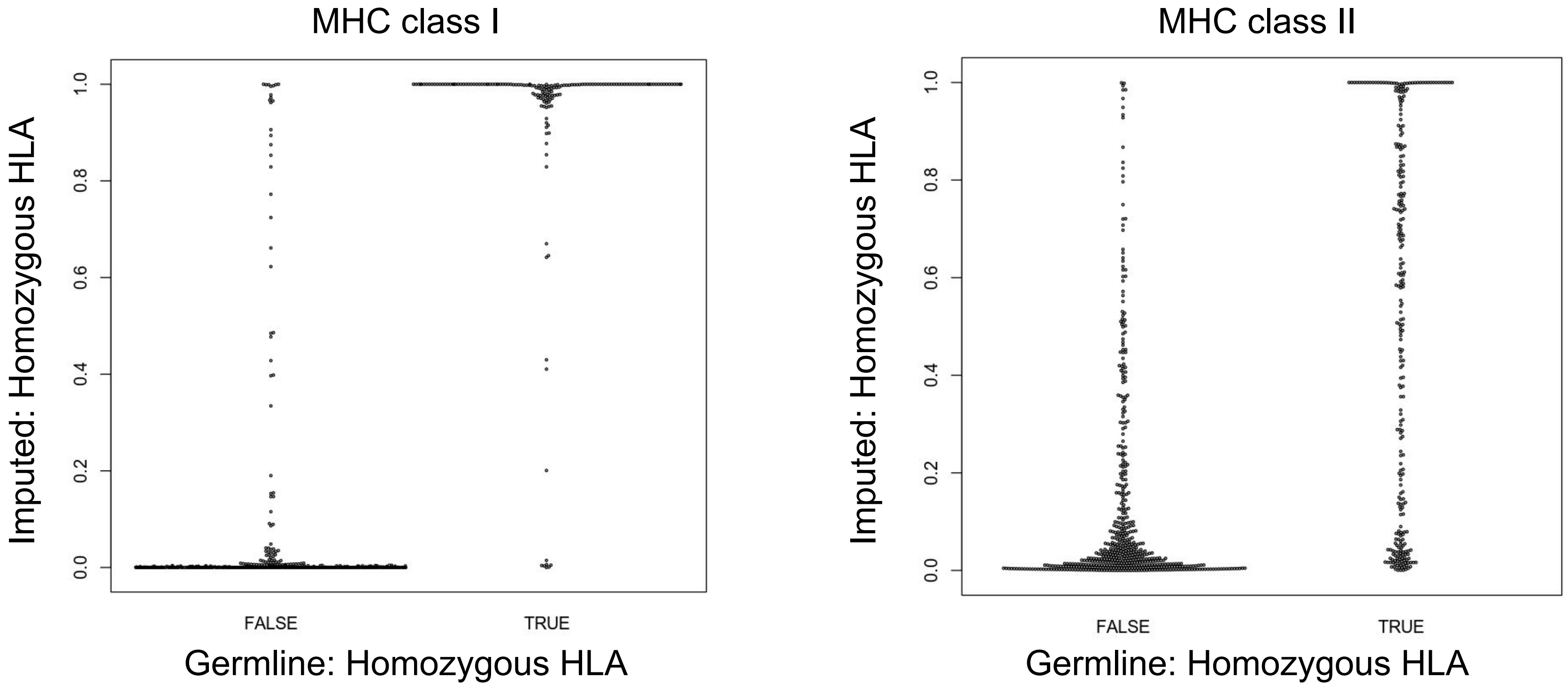

Fig S18: HLA homozygosity calling accuracy. Beehive plot of imputed HLA homozygosity probability (y-axis) versus true HLA homozygosity (x-axis) shown for MHC class I and class II. Homozygosity is defined as being homozygous for at least one allele.

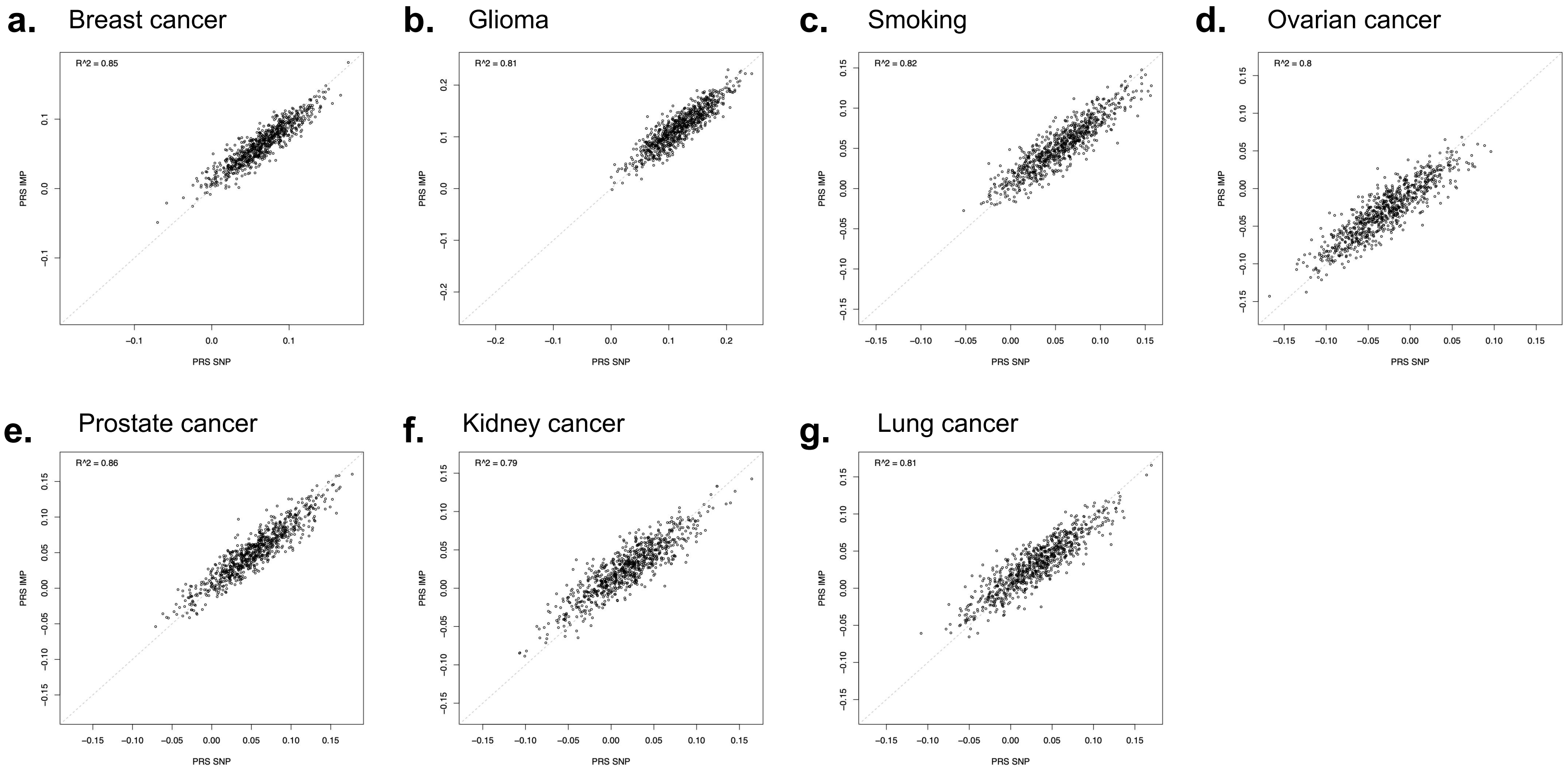

Fig S19: PRS imputation accuracy. Scatter plot of genotyped (x-axis) and imputed (y-axis) PRS scores across individuals (points). Scores used were: (a) breast cancer; (b) glioma; (c) smoking; (d) ovarian cancer; (e) prostate cancer; (f) kidney cancer; (g) lung cancer.

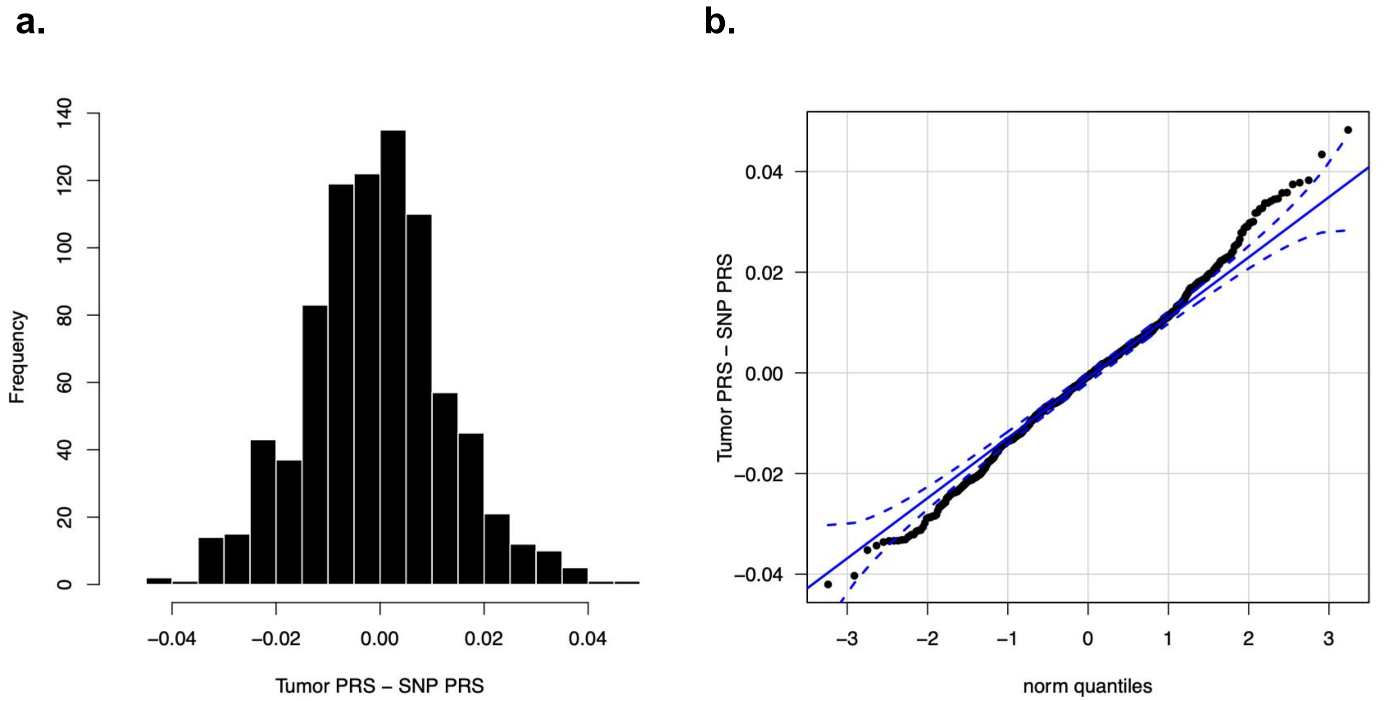

Fig S20: Breast PRS error. As also shown in Figure 4. Histogram (**a**) and QQ-plot (**b**) of tumor imputed minus SNP PRS error.

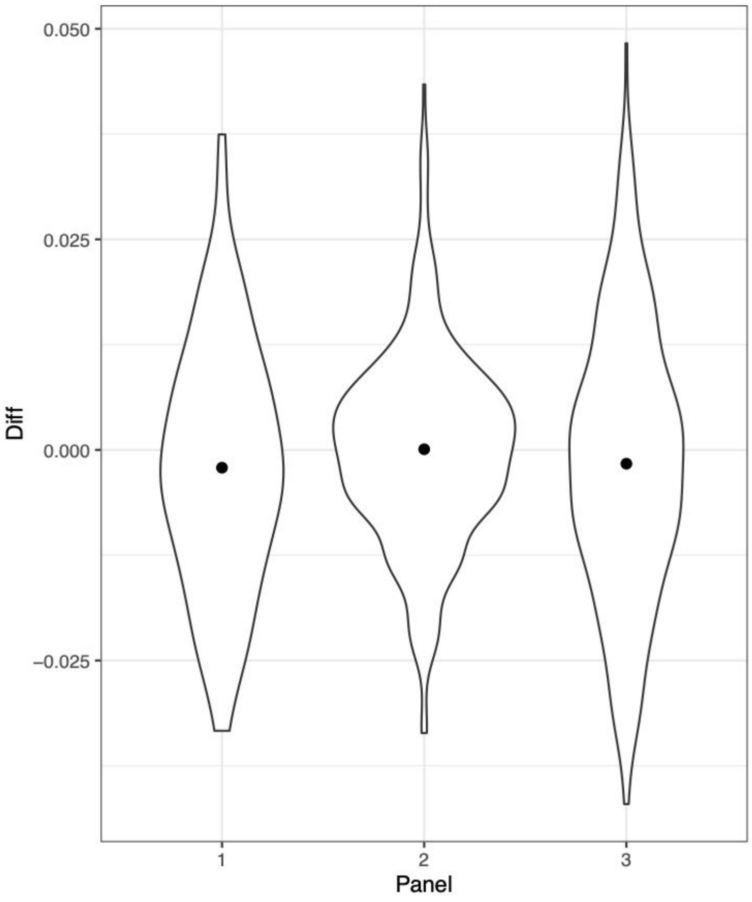

Fig S21: PRS mean error by panel. Violin plot of PRS error (genotyped minus imputed) across three versions of OncoPanel.

Table S1: Association of somatic features with imputation error. Estimates for a joint linear regression.

Feature	Effect size	s.e.	T-statistic	P-value
Coverage	-1.8E+00	4.7E-02	-37.7	**8.5E-177**
Metastasis	1.8E-03	1.4E-03	1.3	2.1E-01
TMB	3.0E-05	8.2E-05	0.4	7.1E-01
Panel 2	-3.2E-03	2.0E-03	-1.6	1.1E-01
Panel 3	7.5E-02	2.3E-03	32.2	**9.0E-145**
Panel 3.1	4.3E-02	2.7E-03	16.1	**2.8E-50**
Purity	9.5E-05	2.9E-05	3.3	**1.2E-03**

Table S2: Number of somatic SNVs per sample that overlap a common reference panel variant in PCAWG tumor WGS data. For each cancer type in PCAWG, numbers indicate the number of samples of that cancer type; the total number of somatic SNVs that overlap a germline reference panel variant; the total number of somatic SNVs observed; and the number of overlapping SNVs per sample.

Cancer	# Samples	# Overlapping SNVs	# Total SNVs	# Overlapping/sample
BLCA-US	412	12	1180313	0.029
BRCA-US	1021	141	941018	0.138
CESC-US	290	47	763083	0.162
COAD-US	403	80	2060051	0.199
DLBC-US	39	14	58448	0.359
GBM-US	389	3	517357	0.008
HNSC-US	509	6	916749	0.012
KICH-US	67	0	37467	0.000
KIRC-US	362	14	226714	0.039
KIRP-US	279	8	272848	0.029
LAML-US	140	6	59565	0.043
LGG-US	509	7	295137	0.014
LIHC-US	365	14	449912	0.038
LUAD-US	517	7	1456859	0.014
LUSC-US	486	24	1438016	0.049
OV-US	427	16	393323	0.037
PAAD-US	178	63	223520	0.354
PRAD-US	498	0	240974	0.000
READ-US	144	61	484487	0.424
RT-US	35	0	1036	0.000
SARC-US	241	110	230127	0.456
SKCM-US	467	27	3942807	0.058
STAD-US	440	21	1707189	0.048
THCA-US	493	0	94330	0.000
UCEC-US	532	805	6607565	1.513

Table S3: EGFR associations with race and ancestry. (**a**) Association of EGFR somatic mutation carriers with race/ancestry features across three models. Asian ancestry and race exhibited a correlation of >0.9 in this dataset resulting in substantially lower significance of each feature in the combined model due to collinearity. (**b**) EGFR somatic mutation carrier frequency across race/ancestry groups.

a.

Model	Ancestry p-value	Race p-value
EGFR ~ race	-	2.3×10^{-21}
EGFR ~ ancestry	3.5×10^{-22}	-
EGFR ~ ancestry + race	0.001	0.33

b.

Race	All	Asian ancestry	European ancestry
Self-reported White	17%	47%	17%
Self-reported Asian	56%	58%	-
